# Decoding a Gut Commensal Signal: Structural and Immunological Profiling of *Segatella Copri* Lipopolysaccharide

**DOI:** 10.1002/anie.202512947

**Published:** 2025-09-30

**Authors:** Luca De Simone Carone, Giusi Barra, Roberta Cirella, Marcello Ziaco, Marcello Mercogliano, Francesca Olmeo, Emanuela Andretta, Valentina Mazziotti, Carmela Fusco, Giuliana D'Ippolito, Katarzyna Anna Duda, Freda M. Farquharson, Petra Louis, Angelo Fontana, Alba Silipo, Antonio Molinaro, Fabrizio Chiodo, Flaviana Di Lorenzo

**Affiliations:** ^1^ Department of Chemical Sciences University of Naples Federico II via Cinthia, 4 Naples 80126 Italy; ^2^ CEINGE‐Biotecnologie Avanzate Franco Salvatore Via Gaetano Salvatore 486 Naples 80145 Italy; ^3^ Department of Biology University of Naples Federico II via Cinthia, 4 Naples 80126 Italy; ^4^ Bio‐Organic Chemistry Unit Institute of Biomolecular Chemistry CNR Via Campi Flegrei 34 Naples 80078 Italy; ^5^ Research Center Borstel Leibniz Lung Center 23845 Borstel Germany; ^6^ The Rowett Institute University of Aberdeen Foresterhill Aberdeen AB25 2ZD UK; ^7^ Department of Molecular Cell Biology and Immunology Amsterdam UMC Vrije Universiteit Amsterdam Amsterdam 1081 HV The Netherlands

**Keywords:** Cytometry by time‐of‐flight, Gut microbiota, Lipopolysaccharides, *Segatella*/*Prevotella*, Structure‐to‐function relationship

## Abstract

The immunological effects of lipopolysaccharides (LPSs) from gut microbiota remain poorly explored, overshadowed by the longstanding view of LPS as a prototypical pro‐inflammatory molecule. Herein, we report the first comprehensive chemical and immunological characterization of LPS from *Segatella copri* DSM 18205, a prominent member of the human oral and intestinal microbiota. This LPS features a unique chemical architecture, including a mannose‐ and glucose‐rich oligosaccharide (OS) and a highly heterogeneous, hypo‐acylated lipid A domain, as elucidated by advanced mass spectrometry (MS) and nuclear magnetic resonance (NMR) spectroscopy. Functionally, *S. copri* LPS displayed attenuated TLR4 activation and weak pro‐inflammatory activity. Strikingly, high‐dimensional cytometry by time‐of‐flight (CyTOF) revealed a selective preservation of CD14^+^CD16^+^ monocytes, immune subsets typically depleted by canonical enterobacterial LPSs. These findings identify *S. copri* LPS as a chemically and functionally distinct microbial signature, offering new insights into host‐microbiota immune crosstalk and highlighting its potential for microbiome‐informed immunomodulatory strategies.

## Graphical abstract

The lipopolysaccharide (LPS) of *Segatella copri* is a structurally unique, immunomodulatory glycoconjugate. Combining mass spectrometry (MS), nuclear magnetic resonance (NMR), and cytometry by time‐of‐flight (CyTOF), we uncover a rough‐type LPS with reduced TLR4 agonism and selective monocyte effects, further highlighting the key role of LPS chemistry in microbiota‐immune interactions.

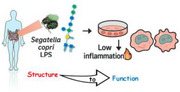

## Introduction

The human gut hosts the most densely populated microbial community in the human body, with hundreds of bacterial species per individual.^[^ ^1^, ^2^ ^]^ Changes in community composition, abundance of certain species, or genetic variation can influence the balance between health and disease and even modulate response to drugs.^[^ ^1^, ^2^ ^]^ As research progresses, the gut microbiota is increasingly recognized not only as a marker of the physiological status but also as a functional player in the realm of precision medicine.^[^ ^3^, ^4^, ^5^ ^]^ This complex microbial ecosystem provides not only a rich metabolic interface with the host but also a vast and largely untapped source of molecular signals that modulate immune responses. Among these, microbial signatures like lipopolysaccharides (LPSs), main glycoconjugates embedded in the outer membrane of Gram‐negative bacteria, have long been recognized as potent immunostimulatory molecules.^[^ ^6^ ^]^ In their smooth‐type form (S‐LPS), they are built up of three moieties, a glycolipid anchor to the membrane (the lipid A), an oligosaccharide (core OS), and a polysaccharide chain (O‐antigen). When the polysaccharide moiety is absent, the terminology used is rough‐type LPS (R‐LPS).^[^ ^7^ ^]^ Traditionally viewed primarily through the lens of their pro‐inflammatory potential, LPSs are now slowly being reconsidered for their broad and context‐dependent roles, especially in the gut, where they are in constant contact with the host immune system.^[^ ^6^, ^8^ ^]^ Indeed, a growing body of evidence suggests that LPSs can differ both chemically and immunologically. As an example, enterobacterial LPSs, such as those from *Escherichia coli* or *Salmonella*, are canonical drivers of endotoxemia and inflammation.^[^ ^6^, ^7^ ^]^ In contrast, we have recently reported several lines of evidence indicating that LPSs from commensal and beneficial bacteria residing in human intestines may exhibit low or even anti‐inflammatory activity.^[^ ^9^, ^10^, ^11^, ^12^, ^13^, ^14^, ^15^ ^]^ These functional differences are rooted in the chemical diversity of these complex molecules, which strongly dictates their immunological properties.^[^ ^6^, ^7^ ^]^ However, despite the centrality of this crucial relationship in host‐microbe interactions, the structural and immunological profiling of LPS from gut‐resident bacteria still remains largely underexplored, and the chemical basis of their divergent host effects is still poorly defined.

*Segatella copri* (formerly known as *Prevotella copri*),^[^ ^16^ ^]^ a prevalent member of the human oral cavity and gastrointestinal tract,^[^ ^17^ ^]^ has been the subject of intense research, being associated with both beneficial and detrimental effects on human health.^[^ ^17^ ^]^ However, the molecular mechanisms by which *S. copri* strains influence host immunity remain mostly undefined, and, more critically, the role of *S. copri* LPS in orchestrating immune responses has only been marginally explored.^[^ ^18^ ^]^ This represents a significant gap of knowledge, as LPS structural and functional properties could provide mechanistic bases for the divergent effects of *S. copri* observed in clinical and preclinical studies. Indeed, mastering the sophisticated language of LPS chemistry requires a concerted chemical and immunological effort, but the reward of understanding and tuning the mechanism of the LPS‐mediated immune response is of outstanding importance in several fields of science.

In this perspective, herein we report the first comprehensive structural elucidation of LPS from *S. copri* DSM 18205, combining advanced mass spectrometry (MS) and nuclear magnetic resonance (NMR) spectroscopy to uncover a previously unreported LPS structure. Our analyses reveal substantial divergence from “canonical” enterobacterial LPS, highlighting unique structural motifs likely responsible for modulating host immune recognition. To complement this chemical characterization, we investigated functional responses of these features using several in vitro human cell models and assessed cytokine release in peripheral blood mononuclear cells (PBMCs). Moreover, to expand the knowledge at the molecular and immunological level, we applied cytometry by time‐of‐flight (CyTOF), which enabled a comprehensive profiling of the immune response elicited by this peculiar LPS. Strikingly, *S. copri* LPS triggered milder pro‐inflammatory responses compared to *E. coli* LPS and uniquely induced differentiation of CD14^+^CD16^+^ monocytes but preserved the intermediate and non‐classical subsets typically depleted by canonical LPSs. This selective effect, absent in classical monocytes and other CD16^+^ cells, highlights *S. copri* LPS as a chemically distinct immunomodulator whose structure‐function relationship offers new insights into the dialogue between the microbiota and the host immune system.

## Results and Discussion

### Structural Characterization of *S. copri* DSM 18205 R‐LPS

LPS from *S. copri* DSM 18205 was isolated from lyophilized bacterial biomass through established protocols (see Note S1).^[^ ^19^ ^]^ The profile of the extract was assessed by SDS‐PAGE followed by silver nitrate gel staining, which revealed the rough nature of the LPS (R‐LPS), i.e., the absence of the polysaccharide portion of the O‐antigen (Figure S1A). The purity of the preparation was verified by complementary assays specifically designed to exclude typical immunostimulatory contaminants, such as phospholipids and lipoproteins/lipopeptides, as confirmed by negative results in SDS‐PAGE with Coomassie blue staining and in the micro‐BCA assay (Figure S1B,C). Monosaccharide composition analysis^[^ ^20^ ^]^ revealed the presence of terminal, 2‐substituted, and 2,6‐disubstituted D‐mannopyranose (D‐Man*p*); terminal, 2‐ and 6‐substituted D‐glucopyranose (D‐Glc*p*); 6‐substituted 2‐amino‐D‐glucopyranose (Glc*p*N); and 4,5‐disubstituted 3‐deoxy‐D‐*manno*‐oct‐2‐ulosonic acid (Kdo). The heterogenous fatty acid content of the lipid A portion was also defined (Figure S1D and Table S1). By combining matrix‐assisted laser desorption/ionization time‐of‐flight MS (MALDI‐TOF MS), electrospray (ESI) MS, tandem MS (MS/MS), and NMR spectroscopy data, we conducted a comprehensive structural elucidation of the R‐LPS. To dissect the lipid A domain, an aliquot of sample was subjected to mild acid hydrolysis, a treatment that selectively cleaves the linkage between the non‐reducing glucosamine of the lipid A portion and the Kdo, which anchors the core oligosaccharide (OS) to the lipid A. The released lipid A was mainly characterized by MALDI‐TOF MS and MS/MS supported by compositional analyses. As for the carbohydrate moiety, structural determination was achieved via NMR investigation of R‐LPS upon full deacylation followed by size‐exclusion chromatography purification. Finally, an in‐depth analysis of intact R‐LPS was also performed by using both ESI‐MS and MALDI‐TOF MS.

**Figure 1:**
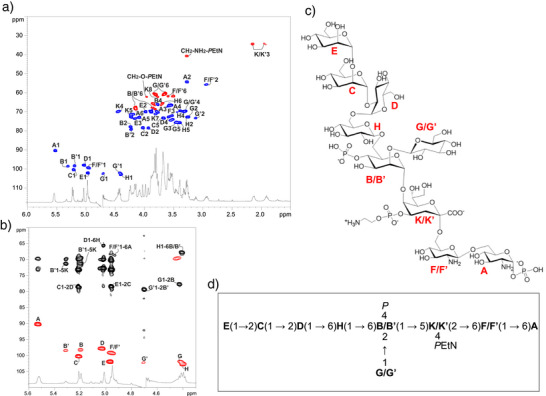
NMR structural analysis of *S. copri* R‐LPS. (600 MHz, 298 K, D_2_O, Table S2). a) Superimposed ^1^H and ^1^H,^13^C HSQC (red and blue) spectra of the core OS obtained after full deacylation of R‐LPS from *S. copri*. Main heteronuclear one‐bond correlations are indicated in the figure. b) Zoom of the overlapped ^1^H, ^1^H, ^13^C HSQC (red) and ^1^H, ^13^C HMBC (black) spectra of the core OS from *S. copri* R‐LPS. Key inter‐residue long‐range correlations involving sugar units are highlighted; residue annotations correspond to those in Table S2. c) and d) Structure of the core OS deduced by NMR spectroscopy, reported using letters as indicated in Table S2.

The ^1^H and ^1^H, ^13^C HSQC NMR spectra of the fully deacylated R‐LPS (Figures 1a and S2) showed the presence of eleven anomeric signals, indicative of eleven spin systems (**A–H**, Figure 1; Table S2), while H‐3 methylene resonances of a Kdo residue (**K/K’**) were also detected in the spectra at *δ*_H_ 2.14/1.93–1.91 ppm and *δ*_C_ 34.4 ppm (Figure 1a; Table S2). The identification of all spin systems was carried out by following the spin connectivity observed in the DQF‐COSY and TOCSY spectra (Figure S3). Simultaneously, carbon atoms were assigned through interpretation of the HSQC spectrum (Figure 1a).^[^ ^21^ ^]^ The anomeric configurations of the monosaccharide units were determined by examining intra‐residue NOE correlations in the ROESY spectrum, along with the ^3^ *J*_H1,H2_ coupling constant values derived from the DQF‐COSY. Additionally, vicinal ^3^ *J*_H,H_ coupling constant values were used to infer the relative configuration of each sugar residue, in turn supported by chemical analyses (see Supporting Information for complete NMR structural discussion; Note S2; Figures S2–S4 and Table S2).

Briefly, the fully deacylated R‐LPS showed a core OS made up of a heptasaccharide composed of one α‐Kdo (**K/K’**), three α‐D‐Man (**B**/**B’**, **C**, and **E**), two β‐D‐Glc (**G/G’** and **H**), and one α‐D‐Glc (**D**), while the two glucosamine residues (α‐D‐GlcN and β‐D‐GlcN) composing the lipid A backbone were identified in spin systems **A** and **F/F’**, respectively. Moreover, the Kdo was found to bear a 2‐aminoethyl phosphate (*P*EtN) unit at its O‐4 position, as proven by the correlation of H‐4 **K/K’** with a signal at −0.75 ppm in the ^31^P,^1^H HSQC spectrum, in turn correlating with methylene proton resonances at *δ*_H_ 3.95 and 3.26 ppm. Moreover, two further monophosphate ester groups with chemical shifts at 3.61 and 4.10 ppm (Table S2) were found and correlated with the anomeric proton signal of the lipid A non‐reducing α‐D‐GlcN **A** (5.52 ppm) and H‐4 of α‐D‐Man **B** (3.77 ppm), respectively. The non‐stoichiometric phosphorylation of α‐D‐Man **B** splits the NMR signals of sugar residues **B**, **G**, **K**, and **F**, resulting in additional spin systems **B’**, **G’**, **K’**, and **F’**. The core OS sequence was elucidated based on inter‐residue NOE correlations detected in the ROESY spectrum (Figure S4), complemented by long‐range correlations observed in the HMBC spectrum (Figure 1b). Briefly, starting from Kdo unit (**K**), this was substituted at the O‐5 position by α‐D‐Man **B**/**B’**, as indicated by the long‐range correlation between the H‐1 signal of **B**/**B’** and the C‐5 signal of **K** (Figure 1b). α‐D‐Man **B**/**B’** was in turn substituted at O‐2 by β‐D‐Glc **G**/**G’** and at O‐6 by β‐D‐Glc **H**, as indicated by the NOE correlation between H‐1 of **G**/**G’** and H‐2 of **B**/**B’** and between H‐1 of **H** and H‐6 of **B**/**B’** (Figure S4), as well as by the related HMBC correlations (Figure 1b). The latter residue **H** showed to bear α‐D‐Glc **D** at its O‐6 position, which in turn carried the α‐D‐Man‐(1→2)‐α‐D‐Man disaccharide (**E** and **C**, respectively) at its O‐6 position. Hence, integration of the results obtained from the NMR analysis enabled the full structural characterization of the carbohydrate moiety from *S. copri* DSM 18205 R‐LPS, as sketched in Figure 1c,d.

MALDI‐TOF MS and MS/MS analyses were performed on the lipid A fraction obtained by mild acid hydrolysis of R‐LPS to determine its structural features. The MALDI‐TOF MS profile (Figure S5A) revealed remarkable heterogeneity among lipid A species, varying in both acylation and phosphorylation states. Moreover, a striking diversity was observed within each acylation group, as evidenced by recurring mass differences of 14 amu, indicative of –(CH_2_)– units and hence corresponding to differences in the acyl chain length. Predominantly, the observed lipid A forms were mono‐phosphorylated tetra‐ and penta‐acylated, consistent with previous observations for the *Prevotella* genus.^[^ ^22^, ^23^ ^]^ Notably, it was also possible to identify minor penta‐acylated lipid A species bearing an additional phosphate group (at around *m/z* 1738, Figure S5A). To validate the structural assignments and rule out any potential artifacts caused by the acid hydrolysis procedure, MALDI‐TOF MS was directly applied to intact bacterial cells. As shown in Figure S5B, this analysis corroborated and strengthened the conclusions drawn from the acid‐hydrolyzed fraction, clearly confirming also the presence of tetra‐ and penta‐acylated lipid A species in their bis‐phosphorylated forms.

**Figure 2:**
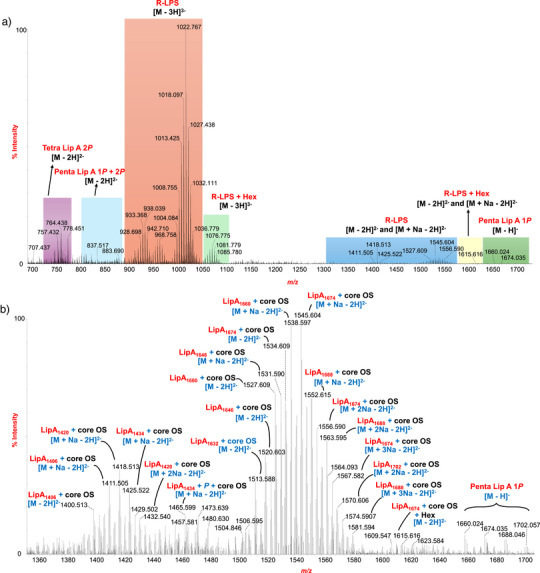
MS structural analysis of *S. copri* R‐LPS. a) Zoom of the negative‐ion ESI mass spectrum of *S. copri* R‐LPS. Colored boxes indicate regions where [M − 3H]^3−^, [M − 2H]^2−^, and [M + Na^n^ − 2H]^2−^ ions corresponding to R‐LPS are found. Colored boxes have also been used to indicate the [M − 2H]^2−^ and [M−H] ions related to lipid A. b) Magnified view of the region where [M − 2H]^2−^ and [M + Na^n^ − 2H]^2−^ ions corresponding to R‐LPS and [M−H] ions related to lipid A are detected.

**Figure 3:**
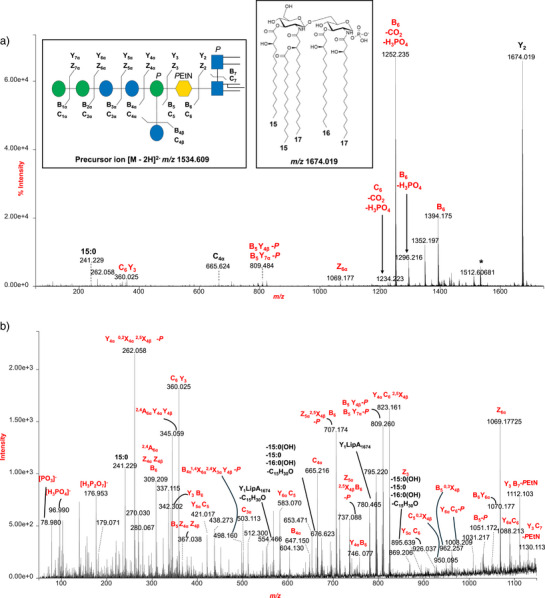
a) Zoom of the negative‐ion MS/MS spectrum of the [M − 2H]^2−^ precursor ion at *m/z* 1534.609 (3070.582 Da) corresponding to full R‐LPS composed of mono‐phosphorylated penta‐acylated lipid A (*m/z* 1674.019, Y_2_ fragment ion) and core OS (*m/z* 1394.175, B_6_ fragment ion). b) Magnified view of the *m/z* range 50–1150. Spectra are the result of the bisection of the R‐LPS, which yielded fragment ions derived from core OS and lipid A as well as hybrid ones containing both lipid and sugar moieties. Fragment ions related to the lipid A moiety are shown in black, while those originating from the saccharide portion are highlighted in red. Lack of C_15_H_30_O is related to a six‐membered ring‐based rearrangement due to an enamine to imine tautomerization that can occur when the *N*‐linked chains (in this case 17:0(3‐OH)) do not carry secondary fatty acids, i.e., they have a free 3‐OH group. The proposed structure for the OS species was drawn using the symbol nomenclature for glycans (SNFG). The nomenclature of glycan fragment ions generated from the cleavage of a glycosidic bond is also reported. Symbols legend: blue circle: glucose; green circle: mannose; blue square: glucosamine; yellow hexagon: Kdo; *P* and *P*EtN stand for phosphate and 2‐aminoethyl phosphate.

A comprehensive structural overview of the R‐LPS was accomplished by ESI‐MS, MALDI‐TOF MS, and tandem MS analyses that were performed on the intact molecule. Direct infusion ESI‐MS analysis of R‐LPS in negative‐ion mode revealed a complex distribution of ions corresponding to both intact R‐LPS species and their characteristic fragment ions related to lipid A and the core OS (see also Note S3 and Table S3). The intact R‐LPS molecules were predominantly observed in the triply charged state ([M − 3H]^3−^), with a major signal detected at *m/z* 1022.767 (3070.582 Da) (Figure 2a), consistent with the full R‐LPS structure comprising a mono‐phosphorylated penta‐acylated lipid A moiety (*m/z* 1674.035) linked to a core OS consisting of six hexoses (Hex), one Kdo, one phosphate group, and one *P*EtN, thus in agreement with NMR analysis. A minor population of [M − 3H]^3−^ ions was also detected at approximately *m/z* 1076.775 (3232.665 Da) (Figure 2a) and was assigned to R‐LPS species carrying an additional Hex residue not identified by NMR, thereby suggesting minor heterogeneity within the core OS structure. A magnified view of the spectrum (Figure 2b) in the *m/z* 1360–1710 range provided detailed insights into the heterogeneity of lipid A and R‐LPS species. Multiple series of doubly charged ([M − 2H]^2−^) ions corresponding to R‐LPS variants were observed, differing in acylation pattern, phosphorylation state, and presence of sodium adducts. Additionally, singly charged ions between *m/z* 1660.024 and 1702.057 were identified as mono‐phosphorylated penta‐acylated lipid A species (Figures 2a,b and S5). Further structural confirmation was obtained through MS/MS analysis of the [M − 2H]^2−^ ion at *m/z* 1534.609 and the [M − 3H]^3−^ ion at *m/z* 1022.767 (3070.582 Da) (Figures 3a,b and S6). In particular, the MS/MS spectrum of the precursor ion at *m/z* 1534.609 revealed a complex fragmentation profile, with signals originating from both the lipid A and the core OS moieties (Figure 3). Prominent peaks at *m/z* 1674.019 and *m/z* 1394.175 were assigned to lipid A and core OS, respectively. However, glycosidic and cross‐ring fragments characteristic of the core region (e.g., Y_3_B_6_, C_4α_, Y_5α_C_6_,_…_)^[^ ^24^ ^]^ could be observed in the *m/z* 50–1150 range, which provided detailed structural information on the glycosidic sequence and branching points of the OS, thus corroborating NMR analysis (Figure 3b).

Likewise, lipid A‐related fragments (labeled in black in Figure 3) were clearly observed and confirmed that the species at *m/z* 1674.019 consists of two glucosamines, one phosphate, two *N*‐linked primary 17:0(3‐OH), one 15:0(3‐OH), and one 16:0(3‐OH) as *O*‐linked primary acyl chains of the non‐reducing and reducing glucosamine, respectively, while 15:0 was the secondary acyl substituent in the acyloxyacylamide moiety of the non‐reducing glucosamine (inset of Figure 3a). The spectrum also includes hybrid fragments containing both lipid and sugar elements (e.g., Y_3_C_7_, Z_3_…), confirming extensive fragmentation across the glycosidic linkage between the core and lipid A.

Of note, Figure 2a also showed significant signals between *m/z* 757.432 and *m/z* 778.451 that were assigned to [M − 2H]^2−^ ions of tetra‐acylated lipid A species bearing two phosphate groups. To further explore this lipid A structural variability and to determine the precise location of the second phosphate group on the glucosamine disaccharide backbone, negative‐ion MS/MS analyses were performed on several precursor ions. This analysis enabled the assignment of the second phosphate group to the non‐reducing glucosamine, as demonstrated by the observation in the MS/MS spectrum of the [M − 2H]^2−^ precursor ion at *m/z* 764.4 (1529.016 Da) (Figure S7) of both the Y_1_− and C_2_ ions at *m/z* 766.478 and *m/z* 778.899 that indicated the presence of two 16:0(3‐OH) and one phosphate on the reducing glucosamine and of one 17:0(3‐OH), one 17:0, and one phosphate on the non‐reducing unit, respectively (see also Note S3). Finally, full R‐LPS also underwent a negative‐ion MALDI‐TOF MS analysis that provided complementary insights into its structural composition (Figure S8) and confirmed the microheterogeneity of the R‐LPS preparation almost fully occurring in the lipid A moiety. In particular, the MALDI‐TOF spectrum clearly displayed distinct ion clusters corresponding to lipid A species that were both mono‐ and bis‐phosphorylated and tetra‐ and penta‐acylated, which were likewise identified as components of the related R‐LPS molecules.

### An In Silico Glimpse of *S. copri* DSM 18205 R‐LPS Biosynthesis

To rationalize the unusual structural features revealed by MS and NMR, we next examined the genome of *S. copri* DSM 18205 to identify loci potentially involved in R‐LPS biosynthesis (a detailed description is reported in Note S4; Figures S9–S12; Tables S4 and S5). The convergence of genomic and chemical evidence, in fact, could provide a coherent framework for LPS biosynthesis and offer insights into how gut symbionts may fine‐tune their LPS architecture to shape host‐microbe interactions. Most of the predicted LPS biosynthesis‐related proteins were encoded within operons, which collectively contain 82 proteins, including both LPS‐related and unrelated ones (Figures S9 and S10; Table S4). For these, annotated Pfam domains^[^ ^25^ ^]^ were identified and visualized (Figure S11). These operons include genes involved in the nine conserved steps of lipid A biosynthesis,^[^ ^26^ ^]^ as well as several operons encoding enzymes responsible for lipid A modification, Kdo biosynthesis, and glycosyltransferase activities.

Through this analysis, we confirmed that the *S. copri* genome encodes only one secondary acyltransferase, consistent with the absence of hexa‐acylated lipid A species and in agreement with previous observations in other Bacteroidetes.^[^ ^27^ ^]^ Moreover, we identified two homologs of the *lpxA* gene in *S. copri* (LK433_RS06380 and LK433_RS13255), whereas in *E. coli* this gene is typically present as a single, essential copy for lipid A biosynthesis.^[^ ^28^ ^]^ The presence of two *lpxA* homologs in *S. copri* is particularly intriguing, given that LpxA functions as a homotrimer and serves as a “molecular ruler” whose acyl chain length selectivity directly shapes the structure of the lipid A domain.^[^ ^29^ ^]^ Notably, the two *S. copri* LpxA homologs retain the catalytic residues and characteristic acyltransferase domains, yet share only ∼45% sequence identity with each other. Structural modelling using AlphaFold3^[^ ^30^, ^31^ ^]^ predicted the formation of stable homotrimeric complexes for each isoform, whereas heterotrimeric assemblies appeared less favorable due to weaker interface stability (see Note S4 and Figure S12). This duality, also observed in other Bacteroidetes (Table S5), may represent an evolutionary adaptation to broaden the substrate range. The presence of functionally distinct LpxA variants could thus account for the incorporation of different acyl chains at the 3‐OH position of glucosamine, providing a plausible molecular basis for the lipid A microheterogeneity observed in MS analyses and directly linking sequence variation to chemical diversity. While based on predictive analyses, the functional implications of these insights merit further experimental investigation.

Following the identification of candidate R‐LPS biosynthetic genes, we mapped their corresponding operons and identified two putative operons (271 and 309; Figure S10) likely involved in core OS biosynthesis. These regions encode multiple glycosyltransferases, spanning the GT2, GT4, GT32, GT90, and GT113 families, consistent with the diverse sugar residues identified by NMR and MS (see Note S4). Likewise, we identified genes likely involved in non‐carbohydrate modifications of the R‐LPS (like phosphate and PEtN addition in the case of *S. copri*), such as genes homologous to *eptA* and *eptB* (LK433_RS03400 and LK433_RS06640) for *P*EtN modifications of lipid A and Kdo, while two additional genes (LK433_RS10260 and LK433_RS10015), related to *lpxE/F/T*, may regulate lipid A phosphorylation through phosphate addition or removal.^[^ ^32^, ^33^ ^]^ Taken together, the genetic repertoire mirrors the unusual structural complexity of *S. copri* R‐LPS revealed experimentally, including *P*EtN‐modified Kdo, mannose phosphorylation, and lipid A heterogeneity, thereby reinforcing the tight connection between genome content and chemical phenotype.

### *S. copri* DSM 18205 R‐LPS TLR‐Mediated Activation Pathway

**Figure 4:**
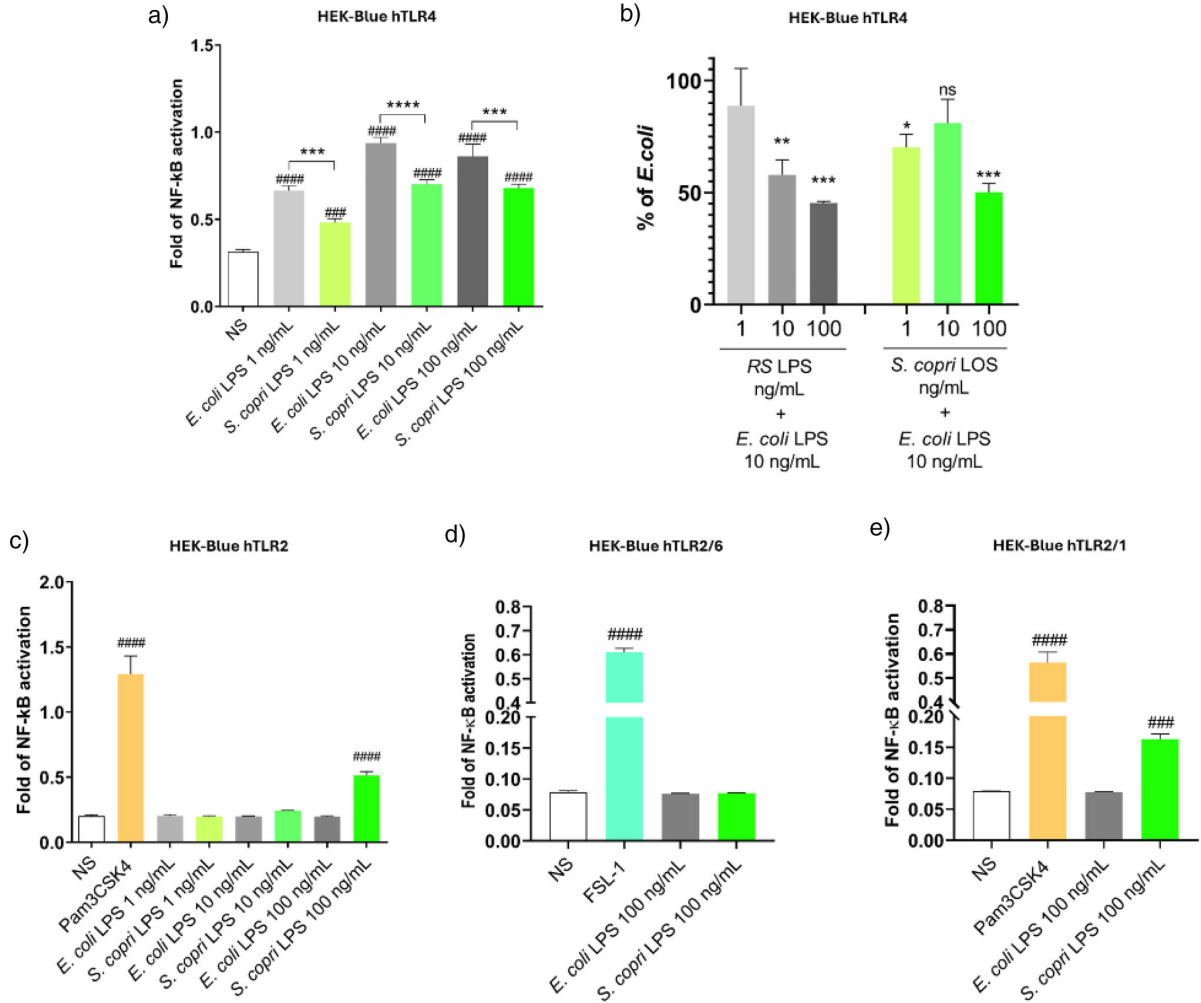
NF‐kB activation in HEK‐Blue cells upon stimulation with *S. copri* R‐LPS. Cells were stimulated for 18 h with either *E*. *coli* LPS or *S. copri* R‐LPS, and NF‐kB activation was quantified by Quanti‐Blue assay (OD 620 nm). The effects of *S. copri* R‐LPS were evaluated on TLR4 a) and TLR2 c). *E. coli* LPS was used as a positive control for TLR4 activation, while treatment with 500 ng mL^−1^ of PAM3CSK4 was employed to verify TLR2 activation. Unstimulated (NS) cells were considered as negative control. b) Competition assay in HEK Blue hTLR4. Cells were pre‐treated with either *R. sphaeroides* (RS) LPS or *S. copri* R‐LPS (1–10–100 ng mL^−1^) for 4 h and then stimulated with 10 ng mL^−1^ *E. coli* LPS for 16 h. NF‐kB activation is expressed as a percentage relative to stimulation with 10 ng mL^−1^ *E. coli* LPS (set as 100%) and shown as mean ± standard deviation. Statistical significance was determined by ordinary one‐way ANOVA (*p‐value < 0.05; **p‐value < 0.01; ***p‐value < 0.001, ****p‐value < 0.0001 versus *E. coli* LPS) a) and b), (^###^ *p*‐value < 0.001 and ^####^ *p*‐value < 0.0001 versus NS) c)–e).

Intrigued by the novel chemical structure and microheterogeneity of *S. copri* R‐LPS, we next evaluated its immunostimulatory potential. Given the well‐established role of lipid A and the core OS in modulating host immune responses, functional assays were performed to assess how *S. copri* R‐LPS interacts with key pattern recognition receptors (PRRs) of the innate immune system, particularly toll‐like receptors (TLRs). To this end, we first employed a panel of cell‐based reporter systems and immune cell lines designed to dissect receptor‐specific activation pathways. These included HEK‐Blue hTLR4 cells, which co‐express human TLR4, MD‐2, and CD14, enabling the selective detection of TLR4‐mediated NF‐κB activation, and HEK‐Blue hTLR2 cells, used to assess potential engagement of the TLR2 pathway. Additionally, differentiated THP‐1 cells were employed as a human macrophage‐like model to evaluate broader innate immune responses, including cytokine release, in a more physiologically relevant context. As shown in Figure 4a, experiments with HEK‐Blue hTLR4 cells demonstrated that *S. copri* R‐LPS induces TLR4‐mediated NF‐κB activation, but at significantly lower levels than *E. coli* LPS across all concentrations tested. This indicated that although recognized by TLR4/MD‐2/CD14, its signaling capacity is markedly attenuated. Remarkably, pre‐incubation with *S. copri* R‐LPS reduced NF‐κB activation by subsequent *E. coli* LPS challenge, demonstrating an inhibitory effect comparable, though slightly weaker, to that of the known TLR4 antagonist *Rhodobacter sphaeroides* LPS (Figure 4b). These results suggest that *S. copri* R‐LPS can compete with pro‐inflammatory LPSs for TLR4 engagement in the gut, thereby modulating the host immune response to stronger LPS stimuli.

Consistent with these findings, treatment of differentiated THP‐1 cells did not alter their viability (Figure S13A) and resulted in modest NF‐κB activation and significantly lower TNF‐α production compared to *E. coli* LPS (Figure S13B), further supporting its weak pro‐inflammatory potential. Finally, since microbiota‐derived LPSs have also been reported to engage TLR2,^[^ ^10^, ^14^ ^]^ we examined this pathway and observed that *S. copri* R‐LPS activated TLR2 signaling only at higher concentrations (100 ng mL^−1^; Figure 4c). As the TLR2 receptor functions as a heterodimer formed with either TLR1 or TLR6, we tested the *S. copri* R‐LPS in a comparative analysis using the hTLR2‐1 (only TLR2 and TLR1 present) and hTLR2‐6 (only TLR2 and TLR6). Interestingly, we observed that *S. copri* R‐LPS triggered a modest activation of the TLR2‐1 heterodimer and not of the TLR2‐6 complex (Figure 4d,e).

### Multiparameter Phenotyping of Human PBMCs Using Mass Cytometry upon Stimulation with *S. copri* DSM 18205 R‐LPS

**Figure 5:**
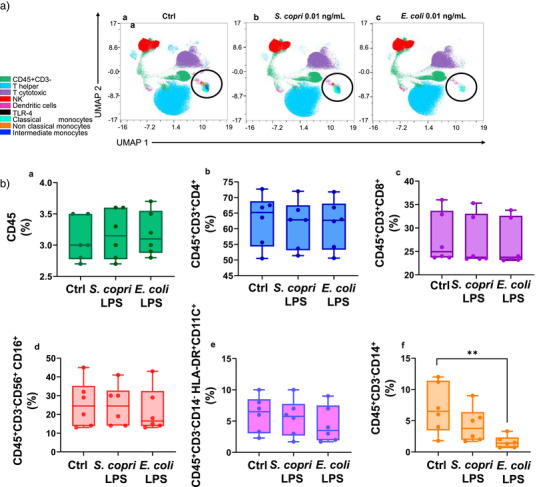
PBMCs immunophenotyping. a) UMAP representation of the main PBMCs populations obtained with CyTOF analysis. Clustering was performed on live cell singlets after quality control and marker selection showed in the gating strategy reported Figure S15 and Table S6. Graphs represent the untreated control (plot a), PBMCs treated with *S. copri* R‐LPS 0.01 ng mL^−1^ (plot b), and with *E. coli* LPS 0.01 ng mL^−1^ (plot c). b) Graphic representation of the main PBMCs subpopulations identified with CyTOF analysis: a) CD3^+^/CD3^−^ ratio; b) T helper; c) T cytotoxic; d) natural killer; e) dendritic cells; and f) monocytes. Graphs represent the mean ± standard deviation of 6 samples. Statistical analysis was performed by the Friedman test with Dunn's post hoc correction. **p‐value < 0.01.

**Figure 6:**
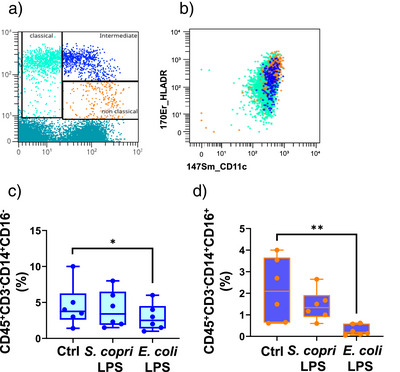
Monocyte subsets: a) Graphic representation of the main monocyte phenotypes: in the Up/Left square the light green population represents the classical monocytes subset (CD14^++^ CD16^−^); in the Up/Right square the blue population represents the CD14^++^CD16^+^ cells known as intermediate monocytes; in the Low/Right square the orange population represents the CD14^+^CD16^+^ subset called non‐classical monocytes. b) Graphical representation of HLA‐DR and CD11c expression levels across the three monocyte subsets; non‐classical (blue) and intermediate (orange) monocytes display the highest expression levels of both markers. c) Mean % of CD45^+^CD14^+^CD16^−^ d) Mean % of CD45^+^CD14^+^CD16^+^ monocytes expression of the six experiments conducted; statistical analysis was performed by the Friedman test with Dunn's post hoc correction. **p‐value < 0.01.

To extend the functional implications of *S. copri* R‐LPS chemical peculiarities, we then assessed its effects on PBMCs, which include a varied population of immune cells mainly composed of lymphocytes (T cells, B cells, and NK cells), monocytes, and dendritic cells.^[^ ^34^ ^]^ PBMCs represent an ex vivo model of the human immune system, enabling the evaluation of complex multicellular immune responses beyond those observed in engineered cell lines.^[^ ^34^ ^]^ To evaluate this response, we measured the production of key cytokines, including TNF‐α, IL‐6, and IL‐10, following stimulation with *S. copri* R‐LPS. These experiments were conducted using the ELLA platform (Bio‐Techne), an automated microfluidic‐based immunoassay system that allows highly sensitive and multiplexed analysis. Overall, *S. copri* R‐LPS induced a milder pro‐inflammatory cytokine profile compared to *E. coli* LPS, with significantly lower levels of TNF‐α and IL‐6 (Figure S14), suggesting a reduced stimulatory capacity on primary immune cells. Likewise, the release of IL‐10 was weaker than that observed in response to *E. coli* LPS at both concentrations tested (Figure S14; 1–10 ng mL^−1^). Of note, *S. copri* R‐LPS was able to induce measurable but moderate cytokine responses in PBMCs, with the most pronounced differences emerging at lower concentrations (1 ng mL^−1^). Given these findings and the desire to better understand the subtle immunomodulatory effects of *S. copri* R‐LPS at physiologically relevant low concentrations, we employed mass cytometry (CyTOF) for an in‐depth characterization of immune cell phenotypes upon stimulation with chemically distinct LPSs. CyTOF, in fact, enables the simultaneous measurement of multiple surface markers, making it ideal to detect fine alterations in complex immune cell populations. Using this highly sensitive approach and applying a multiparameter strategy from systems vaccinology,^[^ ^35^ ^]^ we analyzed PBMC responses to *S. copri* and *E. coli* LPS at low concentrations (0.01 and 0.1 ng mL^−1^), with the aim of uncovering differential capacities to elicit immune activation. We clustered the major immune populations of PBMCs obtained from six healthy donors based on their expression of key surface markers (Table S6 and Figure S15), and as shown in the global UMAP representation (Figure 5a), the most prominent effects of LPS treatments were observed in the monocyte compartment. This was further supported by subset‐specific analysis. In fact, as shown in Figure 5b (panels a–e), neither treatment induced detectable changes in the CD3^+^/CD3^−^ cell ratio, nor were significant effects detected on the CD4^+^ or CD8^+^ T cell populations. Similarly, innate immune cells, such as NK and dendritic cells, appeared unaffected. In contrast, a consistent reduction of monocyte subset CD45^+^CD3^−^CD14^+^ was already detectable after treatment with only 0.01 ng mL^−1^ of *E. coli* LPS (Figure 5b, panel f). This finding aligns with the well‐established role of pro‐inflammatory LPSs in driving monocyte activation and their differentiation into antigen‐presenting cells, a process typically related to CD14 downregulation.^[^ ^36^ ^]^ Intriguingly and in contrast, treatment with 0.01 ng mL^−1^ of *S. copri* R‐LPS led to a modest, non‐significant decrease in CD14 surface expression (Figure 5b, panel f). Notably, the differential effect between the two LPSs became less pronounced at the higher concentration of 0.1 ng mL^−1^, at which both treatments led to a comparable reduction in CD14 expression (Figure S16, panel f). These results suggest that *E. coli* LPS is more potent than *S. copri* R‐LPS in the early triggering of innate immune responses and in particular in the shifts within monocyte cell subsets. To further explore these differential responses and their relevance in immune activation, we analyzed in depth the major monocyte subsets currently known. Monocytes, in fact, are typically classified into three main populations based on CD14 and CD16 surface expression:^[^ ^37^ ^]^ classical (CD14^+^^+^CD16^−^), intermediate (CD14^+^^+^CD16^+^), and non‐classical (CD14^+^CD16^+^). Classical monocytes are primarily involved in homeostatic and regulatory functions, intermediate monocytes are associated with inflammatory responses, and non‐classical monocytes specialize in patrolling and tissue surveillance.^[^ ^38^, ^39^, ^40^, ^41^ ^]^ In our analysis, both *S. copri* and *E*. *coli* LPS mainly impacted CD16^+^ monocyte subsets, including both the intermediate and non‐classical populations. In contrast, the CD16^−^ monocytes were minimally affected, particularly at the lower dose. Importantly, other CD16^+^ cell types, such as NK cells (CD56^+^CD16^+^), remain unaltered upon LPS treatment. This indicates that the observed changes are not merely dependent on CD16 expression alone, but rather on the specific co‐expression of CD14 and CD16 (Figure 5a). Numerous studies have linked the maturation of CD16^+^ monocyte subsets to a more inflammatory antigen‐presenting cell phenotype, in contrast to the more regulatory profile observed for classical monocytes.^[^ ^42^, ^43^ ^]^ Our data further support this observation, as the CD14^+^CD16^+^ monocytes also express elevated levels of activation markers typically related to inflammation, such as CD11c, TLR4, and HLA‐DR (Figure 6a,b).^[^ ^44^ ^]^ As shown in Figure 6c,d, the milder effect of *S. copri* R‐LPS is particularly evident within this specific monocyte subset. Overall, these findings confirm that the immune system differently responds to the two chemically different LPSs even at very low doses. The pro‐inflammatory *E. coli* LPS, even at minimal concentrations, immediately triggers innate immune activation, leading to a reorganization of immune functions that favor pro‐inflammatory responses. In contrast, the microbiota‐derived *S. copri* LPS does not induce the “down‐regulation” of the CD14^+^CD16^+^ population. At lower concentrations, this immune homeostasis is only mildly affected, suggesting the existence of a sort of “threshold,” rooted in its different chemical composition. The stability in the CD14^+^CD16^+^ population provides strong evidence of this phenomenon, as these cells play a central role in immune surveillance. Upon higher LPS concentrations, innate immune alert mechanisms are activated, triggering a response characteristic of early host defense.

## Conclusion

The *Segatella* genus is a predominant component of the gut microbiota in non‐Westernized populations.^[^ ^45^ ^]^ However, whether the presence of *Segatella* spp., and in particular *S. copri* (formerly *P. copri*), exerts beneficial or detrimental effects on human health remains a matter of ongoing debate. Deciphering the chemical and immunological signatures of *S. copri* LPS may be key to resolving the so‐called “*Segatella* paradox” and to identifying microbial features that delineate beneficial from pathogenic host interactions. By integrating high‐resolution MS, NMR spectroscopy, and immune phenotyping, we demonstrate that *S. copri* R‐LPS markedly differs from canonical enterobacterial LPSs in both its molecular architecture and immunological behavior. These findings contribute to the growing appreciation of the chemical and functional diversity of microbiota‐derived LPSs and further underscore the critical importance of resolving LPS chemistry to interpret its biological consequences.^[^ ^6^, ^8^ ^]^

From a structural point of view, *S. copri* LPS features a rough phenotype, i.e., it lacks the O‐antigen polysaccharide moiety and displays a core OS essentially composed of glucose and mannose branched from a Kdo residue substituted at O‐4 by a *P*EtN group. As observed in other Bacteroidetes,^[^ ^23^, ^46^ ^]^ the first sugar linked to the Kdo is an α‐mannose rather than the more conserved L‐*glycero*‐D‐*manno*‐heptose found in enterobacterial LPS core OS.^[^ ^47^ ^]^ Of particular interest is the presence of an α‐1,2‐linked terminal di‐mannose unit at the non‐reducing end of the core. This motif is widely found on the surfaces of diverse microbes, including viruses, fungi, and protozoan parasites, where it plays a pivotal role in host immune engagement. Notably, α‐1,2‐mannose has been shown to interact with C‐type lectin receptors such as DC‐SIGN and mannose‐binding lectin, acting as a molecular “immunological flag” that can promote tolerogenic or context‐dependent immune responses. The localization of this epitope at the non‐reducing end of the *S. copri* core OS tentatively suggests a potential involvement in modulating host immune responses via lectin‐glycan interactions, possibly in parallel with, or independently of, TLR4 signaling, a mechanism previously proposed for other gut‐resident LPS structures.^[^ ^11^, ^48^ ^]^ This intriguing possibility is currently being explored in our laboratory. Strikingly, the lipid A domain displays extensive microheterogeneity, with predominant mono‐phosphorylated tetra‐ and penta‐acylated forms, along with bis‐phosphorylated variants present as minor yet relevant components. Although a deviation from the canonical bis‐phosphorylated hexa‐acylated lipid A of *E. coli* might be expected, *S. copri* lipid A also displays notable differences with closely related *Prevotella/Segatella* species characterized thus far. *P. intermedia* lipid A, e.g., is predominantly mono‐phosphorylated and penta‐acylated, with a simpler and more homogeneous profile.^[^ ^22^ ^]^ Similarly, *P. denticola* was found to be mainly penta‐acylated and carrying *P*EtN on the diglucosamine backbone, with three primary and two secondary acyl chains [one C17(3‐OH), two C16(3‐OH), and two C15:0].^[^ ^23^ ^]^ In line with these structural findings, our in silico analysis identified operons and enzymes predicted to participate in R‐LPS biosynthesis, including multiple glycosyltransferases and lipid A modification enzymes. As an example, the identification of two LpxA isoforms, supported by AlphaFold3 structural predictions, further suggests a possible genomic basis for the observed lipid A heterogeneity. In fact, in contrast to the relatively uniform lipid A species produced by *E. coli*, Bacteroidetes, such as *S. copri*, generate structurally diverse lipid A variants, suggesting that diversification at the level of LpxA may be a key driver of this variability, with potential implications for ad hoc tuning of the host immune response.

In this sense, our structural analysis was complemented with functional assays that revealed an attenuated agonistic activity toward TLR4, justified by its hypo‐acylated and (mostly) hypo‐phosphorylated lipid A structure, and only modest engagement of TLR2 and the TLR2/1 heterodimer at higher concentrations. Likewise, *S. copri* R‐LPS also behaved as a weak activator of the NF‐kB pathway in macrophage‐like THP‐1 cells, inducing minimal TNF‐α release compared to *E. coli* LPS. This suggested that, although *S. copri* R‐LPS can engage TLR4‐dependent signaling, it acts as a weak agonist. This profile may contribute to a mild inflammatory systemic response and, potentially, to a form of finely tuned immune modulation. Consistent with this hypothesis, we have also observed that *S. copri* R‐LPS was able to inhibit TLR4‐dependent NF‐κB activation induced by pro‐inflammatory *E. coli* LPS. This observation suggests that *S. copri* R‐LPS may act as a competitive ligand for the TLR4/MD‐2 complex, potentially dampening host inflammatory responses by occupying the receptor without eliciting a strong activation signal. Accordingly, *S. copri* R‐LPS also elicited only a limited pro‐inflammatory cytokine release in PBMCs, particularly when compared to the robust immune activation triggered by *E. coli* LPS. More intriguingly, mass cytometry analysis enabled high‐dimensional profiling of PBMCs, uncovering the selective effects of *S. copri* R‐LPS on specific monocyte subsets and revealing a distinct immunological footprint for this structurally peculiar glycoconjugate. S*. copri* R‐LPS in fact selectively preserved the CD14^+^CD16^+^ monocyte population, intermediate and non‐classical subsets, critical for immune surveillance and resolution of inflammation. This contrasts with the early depletion of these cells upon *E. coli* LPS exposure, highlighting the attenuated inflammatory signature for *S. copri* R‐LPS while still retaining the capacity to engage host immunity. Besides presenting chemical features typically associated with a low pro‐inflammatory profile, this behavior might reflect the evolutionary adaptation of *S. copri* as a prevalent intestinal commensal, whose LPS may have structural characteristics to which immune cells are already exposed and hence “educated” to their presence. In this view, the structural peculiarities of *S. copri* R‐LPS may not be perceived as a strong alarm signal for systemic innate immune activation, even at higher doses, but instead shape a response, i.e., weak yet still immunologically competent. However, it is important to note that, since PBMCs do not fully reflect the immune cell composition of the intestinal mucosa or its draining lymph nodes,^[^ ^49^ ^]^ these findings should be interpreted as systemic rather than mucosal, and future studies in gut‐relevant models will be required to substantiate this hypothesis.

Together, our findings not only reveal the distinctive chemical identity and immunological behavior of *S. copri* R‐LPS but also establish a framework for evaluating gut microbial LPS as mediators of host‐microbe communication. By linking LPS chemistry to immune phenotypes, this work paves the way for identifying “immunologically silent” or “tuned” LPS structures within the gut microbiota and underscores their potential as next‐generation tools for modulating immunity, developing selective adjuvants, or guiding therapeutic interventions based on microbial chemistry and modulation of the microbiota.

## Supporting Information

The authors have cited additional references within the Supporting Information.^[50–71]^

## Conflict of Interests

The authors declare no conflict of interest.

## Supporting information



Supporting Information

## Data Availability

The data that support the findings of this study are available in the supplementary material of this article.
