# Supplementary material for: Decoding a Gut Commensal Signal: Structural and Immunological Profiling of Segatella Copri Lipopolysaccharide
Source: Angew Chem Int Ed Engl. 2025 Sep 30;64(48):e202512947. doi: 10.1002/anie.202512947 (PMC12643348; doi:10.1002/anie.202512947)
Supplement: Supplementary file 1 — Supporting Information [file ANIE-64-e202512947-s001.docx]

**Supporting Information**

Luca De Simone Carone,^†[a,b,c]^ Giusi Barra,^†[d]^ Roberta Cirella,^[a]^ Marcello Ziaco,^[d]^ Marcello Mercogliano,^[a]^ Francesca Olmeo,^[a]^ Emanuela Andretta,^[a,b]^ Valentina Mazziotti,^[a,b]^ Carmela Fusco,^[d]^ Giuliana D’Ippolito,^[d]^ Katarzyna Anna Duda,^[e]^ Freda M. Farquharson,^[f]^ Petra Louis,^[f]^ Angelo Fontana,^[c,d]^ Alba Silipo,^[a,b]^ Antonio Molinaro,^[a,b]^ Fabrizio Chiodo,^[d,g]^ and Flaviana Di Lorenzo*^[a,b]^

[a] Mr. L. De Simone, Miss R. Cirella, Dr. M. Mercogliano, Miss. F. Olmeo, Dr. E. Andretta, Dr. V. Mazziotti, Prof. A. Silipo, Prof. A. Molinaro, Prof. F. Di Lorenzo
Department of Chemical Sciences
University of Naples Federico II
via Cinthia, 4, 80126, Naples, Italy
E-mail: flaviana.dilorenzo@unina.it

[b] Mr. L. De Simone, Dr. E. Andretta, Dr. V. Mazziotti, Prof. A. Silipo, Prof. A. Molinaro, Prof. F. Di Lorenzo
CEINGE-Biotecnologie Avanzate Franco Salvatore
Via Gaetano Salvatore 486, Naples 80145, Italy

[c] Mr. L. De Simone, Prof. A. Fontana
Department of Biology
University of Naples Federico II
via Cinthia, 4, 80126, Naples, Italy

[d] Dr. G. Barra, Dr. M. Ziaco, Miss C. Fusco, Prof. A. Fontana, Dr. F. Chiodo
Bio-Organic Chemistry Unit
Institute of Biomolecular Chemistry CNR

Via Campi Flegrei 34, 80078 Naples, Italy

[e] Prof. K.A. Duda,

Research Center Borstel Leibniz Lung Center,

23845 Borstel, Germany

[f] Dr. F.M. Farquharson, Dr. L. Petra
The Rowett Institute,

University of Aberdeen,

Foresterhill, Aberdeen AB25 2ZD, United Kingdom

[g] Dr. F. Chiodo
Department of Molecular Cell Biology and Immunology,

Amsterdam UMC, Vrije Universiteit Amsterdam,

Amsterdam 1081 HV, The Netherlands.

**Supporting Tables**

**Table S1.** Fatty acid content of lipid A isolated from *S. copri* DSM 18205 R-LPS.

**Table S2.** ^1^H, ^13^C, and ^31^P chemical shift values of the core OS obtained upon full deacylation of the *S. copri* DSM 18205 R-LPS.

**Table S3.** Negative-ion ESI-MS data and proposed composition of *S. copri* R-LPS.

**Table S4.** List of the genes predicted to be part of the two LPS biosynthesis associated network predicted using STRING.

**Table S5.** List of the loci encoding for LpxA homologs.

**Table S6.** List of immune markers used in CyTOF analysis.

**Supporting Figures**

**Figure S1. A**) Silver staining of SDS-PAGE analysis. **B**) Comassie Billiant Blue staining of SDS-PAGE analysis. **C**) Micro-BCA assay. **D**) Compositional analysis of *S. copri* DSM 18205 R-LPS.

**Figure S2.** (600 MHz, 298 K, D_2_O, Table S2). ^1^H NMR spectrum of the core OS of *S. copri* DSM 18205.

**Figure S3.** (600 MHz, 298 K, D_2_O, Table S2). ^1^H, COSY and TOCSY spectra of the core OS of *S. copri* DSM 18205.

**Figure S4.** (600 MHz, 298 K, D_2_O, Table S2). ^1^H, TOCSY and ROESY spectra of the core OS of *S. copri* DSM 18205.

**Figure S5.** MALDI-TOF MS spectra of the lipid A obtained from mild acid hydrolysis of R-LPS (**A**) and directly on bacterial pellet (**B**) of *S. copri* DSM 18205.

**Figure S6.** Zoom of the negative-ion ESI MS/MS of [M-3H]^3−^ ion at *m/z* 1022.767.

**Figure S7.** Zoom of the negative-ion ESI MS/MS of [M-2H]^2−^ ion at *m/z* 764.4.

**Figure S8.** Negative-ion MALDI-TOF MS spectrum of intact R-LPS from *S. copri* DSM 18205.

**Figure S9.** Functional protein association networks based on STRING.

**Figure S10.** Schematic representation of the operons containing one or more gene predicted to be involved in LPS biosynthetic processes.

**Figure S11.** Predicted Pfam domains for proteins encoded by operons predicted to harbor one or more LPS biosynthesis genes included in the STRING-derived functional networks.

**Figure S12.** *In silico* prediction of the three-dimensional structure of the two LpxA isoforms.

**Figure S13.** Stimulation of THP-1 cells with *S. copri* DSM 18205 R-LPS and *E. coli* LPS.

**Figure S14.** Cytokine release in PBMCs stimulated with *S. copri* DSM 18205 R-LPS and *E. coli* S-LPS.

**Figure S15.** Gating strategy for CyTOF analysis.

**Figure S16.** PBMCs immunophenotyping.

**Supporting notes**

**Supporting note 1.** Methods.

**Supporting note 2.** NMR characterization of the core OS from *S. copri* DSM 18205 R-LPS.

**Supporting note 3.** ESI-MS and MS/MS characterization of the R-LPS from *S. copri* DSM 18205.

**Supporting note 4.** Bioinformatic analysis of genes encoding for *S. copri* DSM 18205 R-LPS biosynthesis.

**Table S1.** Fatty acid composition of lipid A from *S. copri* DSM 18205 was assessed by GC-MS. All detected 3-hydroxy fatty acids exhibited (*R*) absolute configuration. For *anteiso*-branched acyl chains, the configuration is tentatively assigned as (*S*), based on its reported prevalence in bacteria, although definitive assignment has yet to be established.

| *Fatty acid composition* |
| --- |
| *iso* and *anteiso* pentadecanoic acid (*i*15:0 and *a*15:0)  pentadecanoic acid (15:0) |
| hexadecanoic acid (16:0) |
| 3-hydroxytetradecanoic acid [14:0 (3-OH)] |
| *iso* and *anteiso* heptadecanoic acid (*i*17:0 and *a*17:0)) |
| *iso* 3-hydroxypentadecanoic acid [*i*15:0 (3-OH)  3-hydroxypentadecanoic acid [15:0 (3-OH)]  3-hydroxyhexadecanoic acid [16:0 (3-OH)] |
| *iso* and *anteiso* 3-hydroxyheptadecanoic acid [*i*17:0 (3-OH) and *a*17:0 (3-OH)]  3-hydroxyheptadecanoic acid [17:0 (3-OH)] |

**Table S2.** ^1^H, ^13^C, and ^31^P chemical shift values of the core OS obtained upon full deacylation of the *S. copri* DSM 18205 R-LPS.

|  | **1** | **2** | **3** | **4** | **5** | **6** | **7** | **8** |
| --- | --- | --- | --- | --- | --- | --- | --- | --- |
| **A**  6-α-GlcN1*P* | 5.52 | 3.26 | 3.79 | 3.31 | 4.08 | 4.14/3.83 |  |  |
|  | *90.2* | *54.2* | *69.6* | *69.7* | *72.9* | *68.1* |  |  |
|  | ^31^P **3.61** |  |  |  |  |  |  |  |
| **C**  2-α-Man | 5.20 | 4.02 | 3.86 | 3.58 | 3.66 | 3.80/3.65 |  |  |
|  | *100.3* | *78.3* | *69.8* | *66.7* | *73.1* | *60.8* |  |  |
|  |  |  |  |  |  |  |  |  |
| **B/B’**  2,6-α-Man4*P* | 5.31/5.19 | 4.22/4.22 | 3.87/3.88 | 3.57/3.77 | 3.76 | 4.15/3.85 |  |  |
|  | *98.5/98.2* | *79.2/77.8* | *69.8* | *66.8/66.3* | *70.3* | *67.8* |  |  |
|  |  |  |  | ^31^P **4.10** |  |  |  |  |
| **D**  2-α-Glc | 5.02 | 3.93 | 3.88 | 3.58 | 3.78 | 3.79 |  |  |
|  | *98.0* | *78.5* | *69.4* | *72.6* | *70.1* | *61.3* |  |  |
|  |  |  |  |  |  |  |  |  |
| **E**  *t*-α-Man | 4.96 | 3.97 | 4.15 | 3.54 | 3.77 | 3.79/3.66 |  |  |
|  | *102.0* | *69.8* | *73.4* | *66.6* | *70.1* | *60.8* |  |  |
|  |  |  |  |  |  |  |  |  |
| **F/F’**  6-β-GlcN | 4.95/4.92 | 2.93/2.92 | 3.52 | 3.44 | 3.54 | 3.51/3.59 |  |  |
|  | *99.3* | *55.5* | *72.1* | *70.0* | *74.4* | *61.7* |  |  |
|  |  |  |  |  |  |  |  |  |
| **G/G’**  *t*-β-Glc | 4.70/4.42 | 3.12/3.25 | 3.51 | 3.29 | 3.46 | 3.83/3.63 |  |  |
|  | *102.3/102.0* | *73.1/72.6* | *74.0* | *69.5* | *75.3* | *60.3* |  |  |
|  |  |  |  |  |  |  |  |  |
| **H**  6-β-Glc | 4.40 | 3.25 | 3.51 | 3.39 | 3.39 | 3.84/3.65 |  |  |
|  | *102.8* | *72.6* | *73.9* | *69.4* | *75.5* | *65.6* |  |  |
|  |  |  |  |  |  |  |  |  |
| **KK’**  5-α-Kdo4*P*EtN | ----- | ----- | 2.14/1.93  2.14/1.91 | 4.44 | 4.21/4.22 | 3.67 | 3.81 | 3.95/3.77 |
|  | *176.9* | *99.8* | *34.4* | *69.7* | *71.5/72.4* | *73.0* | *70.9* | *62.1* |
|  |  |  |  | ^31^P **-0.75** |  |  |  |  |
| *P*EtN | 3.95 | 3.26 |  |  |  |  |  |  |
|  | *62.1* | *40.2* |  |  |  |  |  |  |

**Table S3.** Negative-ion ESI MS data and proposed composition of *S. copri* R-LPS. Core OS is composed of six hexoses (monoisotopic mass 162.0528), one Kdo (monoisotopic mass 220.0583), one *P*EtN (monoisotopic mass 123.0087) and one phosphate (monoisotopic mass 79.9663).

| **Observed ion (*m/z*)** | | | | | | **Molecular mass (Da)** | | **Proposed composition** |
| --- | --- | --- | --- | --- | --- | --- | --- | --- |
| (M+Na-2H)^2-^ | (M+2Na-2H)^2-^ | (M+3Na-2H)^2-^ | (M-2H)^2-^ | (M-3H)^3-^ |  | Observed  (average) | Calculated |  |
| 1411.505 | 1422.497 | - | 1400.513 | 933.368 |  | 2802.309 | 2802.352 | **LipA_1406_ + core OS** |
| 1418.513 | 1429.502 | - | 1407.515 | 938.039 |  | 2816.319 | 2816.363 | **LipA_1420_ + core OS** |
| 1425.522 | 1436.517 | - | - | 942.710 |  | 2830.098 | 2830.373 | **LipA_1434_ + core OS** |
| 1465.599 | - | - | 1453.598 | 968.758 |  | 2909.578 | 2910.339 | **LipA_1434_ + *P* + core OS** |
| - | 1504.846 | - | 1480.630 | 987.383 |  | 2964.725 | 2964.405 | **LipA_1406_ + core OS**  **+ Hex** |
| 1524.583 | - | - | 1513.588 | 1008.755 |  | 3028.891 | 3028.551 | **LipA_1632_ + core OS** |
| 1531.590 | - | - | 1520.603 | 1013.425 |  | 3042.909 | 3042.561 | **LipA_1646_ + core OS** |
| 1538.597 | 1549.594 | - | 1527.609 | 1018.097 |  | 3056.869 | 3056.571 | **LipA_1660_ + core OS** |
| 1545.604 | 1556.590 | 1567.582 | 1534.609 | 1022.767 |  | 3070.268 | 3070.582 | **LipA_1674_ + core OS** |
| 1552.615 | 1563.595 | 1574.591 | 1542.104 | 1027.438 |  | 3084.444 | 3084.592 | **LipA_1688_ + core OS** |
| 1559.609 | 1570.606 | 1581.594 | - | 1032.111 |  | 3097.771 | 3098.602 | **LipA_1702_ + core OS** |
| - | - | - | 1615.616 | 1076.775 |  | 3233.298 | 3232.665 | **LipA_1674_ + core OS**  **+ Hex** |

**Table S4.** Genes predicted to be part of the two LPS biosynthesis associated networks identified using STRING. For each gene, the corresponding locus tag and the NCBI protein ID (from the genome used in this study) are provided. COGs were predicted using the Operon Mapper web server, while Pfam domains were retrieved by submitting the protein sequences to the InterProScan web service. GO terms and KEGG pathway identifiers were obtained via STRING analysis.

| **Locus Tag** | **Protein ID** | **COG** | **PFAM** | **GO** | **KEGG** | **Description** |
| --- | --- | --- | --- | --- | --- | --- |
| LK433_RS10260 | WP_006846729.1 | COG0671 | PF01569 | GO:0009103 | Map00550 | [I] Membrane-associated phospholipid phosphatase; PAP2 superfamily |
| LK433_RS02395 | WP_006848238.1 | COG0859 | PF01075 | GO:0009103 | Map00550 | [M] ADP-heptose:LPS heptosyltransferase; GT9 |
| LK433_RS00645 | WP_040553634.1 | COG1519 | PF04413 | GO:0009103 | map00540 | [M] 3-deoxy-D-manno-octulosonic-acid transferase |
| LK433_RS08545 | WP_006848677.1 | COG1778 | PF00702 | GO:0009103 | map00540 | [R] Low specificity phosphatase (HAD superfamily); haloacid dehalogenase-like hydrolase |
| LK433_RS12355 | WP_006846279.1 | COG1663 | PF02606 | GO:0009245 | map00540 | [M] Tetraacyldisaccharide-1-P 4'-kinase; Tetraacyldisaccharide-1-P 4'-kinase |
| LK433_RS10210 | WP_006846719.1 | COG1212 | PF02348 | GO:0009103 | map00540 | [M] CMP-2-keto-3-deoxyoctulosonic acid synthetase; Cytidylyltransferase |
| LK433_RS06390 | WP_006847231.1 | COG1044 | PF04613 | GO:0009245 | map00540 | [M] UDP-3-O-[3-hydroxymyristoyl] glucosamine N-acyltransferase (LpxD) |
| LK433_RS06385 | WP_117691601.1 | COG0774 | PF03331  PF07977 | GO:0009245 | map00540 | [M] UDP-3-O-acyl-N-acetylglucosamine deacetylase; UDP-3-O-acyl N-acetylglycosamine deacetylase/ FabA-like domain |
| LK433_RS02745 | WP_006848120.1 | COG1560 | PF03279 | GO:1903509 | map00540 | [M] Lauroyl/myristoyl acyltransferase; Bacterial lipid A biosynthesis acyltransferase |
| LK433_RS11860 | WP_208854995.1 | COG2877 | PF00793 | GO:0009103 | map00540 | [M] 3-deoxy-D-manno-octulosonic acid (KDO) 8-phosphate synthase; DAHP synthetase I family |
| LK433_RS00920 | WP_006847573.1 | COG2908 | - | GO:0009245 | map00540 | [S] Uncharacterized protein conserved in bacteria |
| LK433_RS06010 | -  (LpxB) | COG0763 | PF02684 | GO:0009245 | map00540 | [M] Lipid A disaccharide synthetase; Lipid-A-disaccharide synthetase |
| LK433_RS06380 | WP_006847229.1 | COG1043 | PF13720  **PF00132** | GO:0009245 | map00540 | [M] Acyl-[acyl carrier protein]--UDP-N-acetylglucosamine O-acyltransferase;  **Bacterial transferase hexapeptide (six repeats)** |
| LK433_RS13255 | WP_006849066.1 | COG1043 | PF13720  **PF00132** | GO:0009245 | map00540 | [M] Acyl-[acyl carrier protein]--UDP-N-acetylglucosamine O-acyltransferase; **Bacterial transferase hexapeptide (six repeats)** |
| LK433_RS03400 | WP_006847975.1 | COG2194 | PF00884  PF08019 | GO:0009103 | map00540 | [R] Predicted membrane-associated, metal-dependent hydrolase; Sulfatase/Phosphoethanolamine transferase EptA/EptB |
| LK433_RS06640 | WP_006847287.1 | COG2194 | PF00884  PF08019 | GO:0009103 | map00540 | [R] Predicted membrane-associated, metal-dependent hydrolase; Sulfatase/Phosphoethanolamine transferase EptA/EptB |
| LK433_RS10015 | WP_006846680.1 | COG0671 | PF01569 | GO:0009103 | **-** | [I] Membrane-associated phospholipid phosphatase; PAP2 superfamily |
| LK433_RS01175 | WP_006847625.1 | COG0457 | - | GO:1903509 | **-** | [R] FOG: TPR repeat |
| LK433_RS11500 | WP_006846995.1 | COG1408 | PF00149 | GO:0009245 | **-** | [R] Predicted phosphohydrolases; Calcineurin-like phosphoesterase |
| LK433_RS11925 | WP_006846430.1 | COG3206 | PF02706 | GO:0009103 | **-** | [M] Uncharacterized protein involved in exopolysaccharide biosynthesis, Chain length determinant protein |
| LK433_RS06490 | WP_006847251.1 | COG1408 | PF00149 | GO:0009245 | **-** | [R] Predicted phosphohydrolases; Calcineurin-like phosphoesterase |
| LK433_RS10640 | WP_006846801.1 | ROG8224 | - | GO:0009103 | **-** | NA |
| LK433_RS10635 | WP_006846800.1 | ROG8224 | PF02706 | GO:0009103 | **-** | NA: Chain length determinant protein |
| LK433_RS11865 | WP_006846386.1 | COG0794 | PF00571  PF01380 | **-** | map00540 | [M] Predicted sugar phosphate isomerase involved incapsule formation; CBS domain/SIS domain |
| LK433_RS03340* | WP_006847988.1 | COG3475 | PF04991 | **-** | map00540 | [M] LPS biosynthesis protein; LicD family |
| LK433_RS02735 | WP_006848122.1 | ROG2301 | PF06293* | **-** | map00540 | Lipopolysaccharide kinase (Kdo/WaaP) family |
| LK433_RS02705* | WP_006848130.1 | ROG2301 | PF06293* | **-** | map00540 | Lipopolysaccharide kinase (Kdo/WaaP) family |
| LK433_RS02660* | WP_006848139.1 | COG3344 | PF08388 | **-** | map00540 | [L] Retron-type reverse transcriptase; Group II intron, maturase-specific domain |
| LK433_RS02655* | WP_006848140.1 | COG3344 | PF00078 | **-** | map00540 | [L] Retron-type reverse transcriptase; Reverse transcriptase (RNA-dependent DNA polymerase) |
| LK433_RS02410* | WP_006848234.1 | COG0463 | PF00535 | **-** | map00540 | [M] Glycosyltransferases involved in cell wall biogenesis; Glycosyl transferase family 2 |
| LK433_RS14465* | WP_006848636.1 | COG0463 | PF00535 | **-** | map00540 | [M] Glycosyltransferases involved in cell wall biogenesis; Glycosyl transferase family 2 |
| LK433_RS04725 | WP_006849462.1 | COG0279 | PF01380 | **-** | map00540 | [G] Phosphoheptose isomerase; SIS domain |
| LK433_RS04720 | WP_006849463.1 | COG2605 | PF08544  PF00288 | **-** | map00540 | [R] Predicted kinase related to galactokinase and; GHMP kinases C terminal/ GHMP kinases N terminal domain |
| LK433_RS10425 | WP_040552619.1 | NA | - | **-** | map00540 | NA |
| LK433_RS02815 | WP_082231245.1 | ROG3849 | PF19573 | **-** | map00540 | NA; Domain of unknown function (DUF6089) |
| LK433_RS11575* | WP_228023605.1 | COG3344 | PF08388  PF00078 | **-** | map00540 | [L] Retron-type reverse transcriptase; Group II intron, maturase-specific domain/Reverse transcriptase (RNA-dependent DNA polymerase) |
| LK433_RS08635 | WP_006848659.1 | COG4660 | PF02508 | **-** | map00540 | [C] Predicted NADH:ubiquinone oxidoreductase, subunit RnfE; Rnf-Nqr subunit, membrane protein |

**Table S5.** List of loci encoding LpxA homologs in *S. copri* DSM 18205, *Bacteroides fragilis* NCTC 9343, *B. vulgatus* ATCC 8482, and *B. thetaiotaomicron* VPI-5482. Sequence homology was assessed using BLASTP on the NCBI website. All genes were predicted to belong to COG1043 ([M] Acyl-[acyl carrier protein]-UDP-*N*-acetylglucosamine *O*-acyltransferase) by submitting the corresponding genomes to the Operon Mapper web server.

| **Species** | **Locus lpxA** | **Locus lpxA_2** | **% Identity against *E. coli* LpxA (LpxA)** | **% Identity against *E. coli* LpxA (LpxA_2)** |
| --- | --- | --- | --- | --- |
| *S. copri* | LK433_RS06380  (WP_006847229.1) | LK433_RS13255  (WP_006849066.1) | 43.89% | 30.35% |
| *B. vulgatus* | BVU_RS00505 | BVU_RS01220 | 41.98% | 32.81% |
| *B. thetaiotaomicron* | BT_RS21210 | BT_RS16890 | 41.98% | 35.02% |
| *B. fragilis* | BF9343_RS03875 | BF9343_RS00690 | 41.60% | 35.41% |
| *E. coli* | IEU92_RS00905 | - | 100.00% | - |

**Table S6.** Phenotypic characterization of major peripheral blood mononuclear cell (PBMCs) subsets identified by mass cytometry (CyTOF). The table summarizes the main immune markers used to delineate each corresponding cell cluster.

| **SUBSET** | **MARKERS** |
| --- | --- |
| T helper | CD45^+^ CD3^+^ CD4^+^ |
| T cytotoxic | CD45^+^ CD3^+^ CD8^+^ |
| NK | CD45^+^CD3^-^CD56^+^CD16^+^ |
| Dendritic cells | CD45^+^CD3^-^CD14^-^ HLADR^+^CD11C^+^ |
| Monocytes | CD45^+^CD3^-^CD14^+^ |
| Classical monocytes | CD45^+^CD3^-^CD14^++^CD16^-^ |
| Intermediate monocytes | CD45^+^CD3^-^CD14^++^ CD16^+^ |
| Non classical monocytes | CD45^+^CD14^+^CD16^+^ |

**Supporting Figures**

**Figure S1.** (**A**) Silver and (**B**) Coomassie Brilliant Blue staining of SDS-PAGE of *S. copri* R-LPS. In the silver stained SDS-PAGE gel, S-LPS from *Salmonella typhimurium*SH 2201 (4 and 8 µL) (Lanes 3-4) was used as a benchmark; 4 μL (Lane 1) and 8 μL (Lane 2) of 1 mg/mL solution of *S. copri* R-LPS were loaded on the gel. In the Coomassie Brilliant Blue stained SDS-PAGE gel bovine serum albumin (BSA) (8 µL) (Lane 5) and BLUeye Prestained Protein Ladder (2 µL) (Lane 1) were used as references. 4 μL (Lane 2), 8 μL (Lane 3) and 16 μL of 1 mg/mL solution of *S. copri* R-LPS were loaded on the gel. (**C**) Micro BCA™ Protein Assay performed in duplicate in presence of 2 % SDS. BSA at various concentrations has been used as standard. The graph shows the normalized optical density (OD) over the background signal from water samples. (**D**) GC-MS chromatogram profile of the acetylated methyl glycoside (AMG) and methyl ester derivatives of monosaccharides and fatty acids respectively of *S. copri* R-LPS. Man: Mannose; Glc: Glucose; GlcN: glucosamine; Kdo: 3-deoxy-D-*manno*-oct-2-ulosonic acid.

**Figure S2.** (600 MHz, 298 K, D_2_O, Table S2). ^1^H NMR spectrum of the core OS of *S. copri* DSM 18205 R-LPS.

**Figure S3.** (600 MHz, 298 K, D_2_O, Table S2). Zoom of the overlapped ^1^H, COSY (light blue and pink) and TOCSY (black) spectra of the core OS of *S. copri* DSM 18205. Numbering of sugar residues is as reported in Table S2.

**Figure S4.** (600 MHz, 298 K, D_2_O, Table S2). Zoom of the ^1^H, TOCSY (black) and ROESY (violet) spectra of the core OS of *S. copri* DSM 18205 R-LPS. Numbering of sugar units is as listed in Table S2.

**Figure S5.** MALDI-TOF MS spectra of the lipid A from *S. copri* R-LPS obtained through mild acid hydrolysis (**A**) and directly on bacterial pellets (**B**). “+*P*” is indicative of the *bis*-phosphorylated lipid A species.

**Figure S6.** Zoom of the negative-ion ESI MS/MS of [M-3H]^3−^ ion at *m/z* 1022.767 (3070.582 Da) related to intact R-LPS from *S. copri* composed of a *mono*-phosphorylated penta-acylated lipid A species (1675.2 Da) and the core OS composed of six hexoses, one Kdo, one PEtN, and one phosphate (*P*). In the inset the zoom of the 2D Heat Map of *m/z* versus drift time (bins) where the signals referred to lipid A and core OS are revealed. The color scale reflects the intensities from the most intense yellow to the less intense blue.

**Figure S7.** Zoom of the negative-ion ESI MS/MS of [M-2H]^2−^ ion at *m/z* 764.438 (1529.016 Da) taken as a representative of *bis*-phosphorylated tetra-acylated lipid A species from *S. copri* R-LPS

**Figure S8.** Negative-ion MALDI-TOF MS spectrum, recorded in reflectron mode, of the intact R-LPS from *S. copri*. Tetra-and Penta Lip A indicates the degree of acylation. “1*P*” and “2*P*” is indicative of the *mono*- and *bis*-phosphorylated lipid A species. The zoom of the region containing peaks attributed to R-LPS is reported in the inset.

**
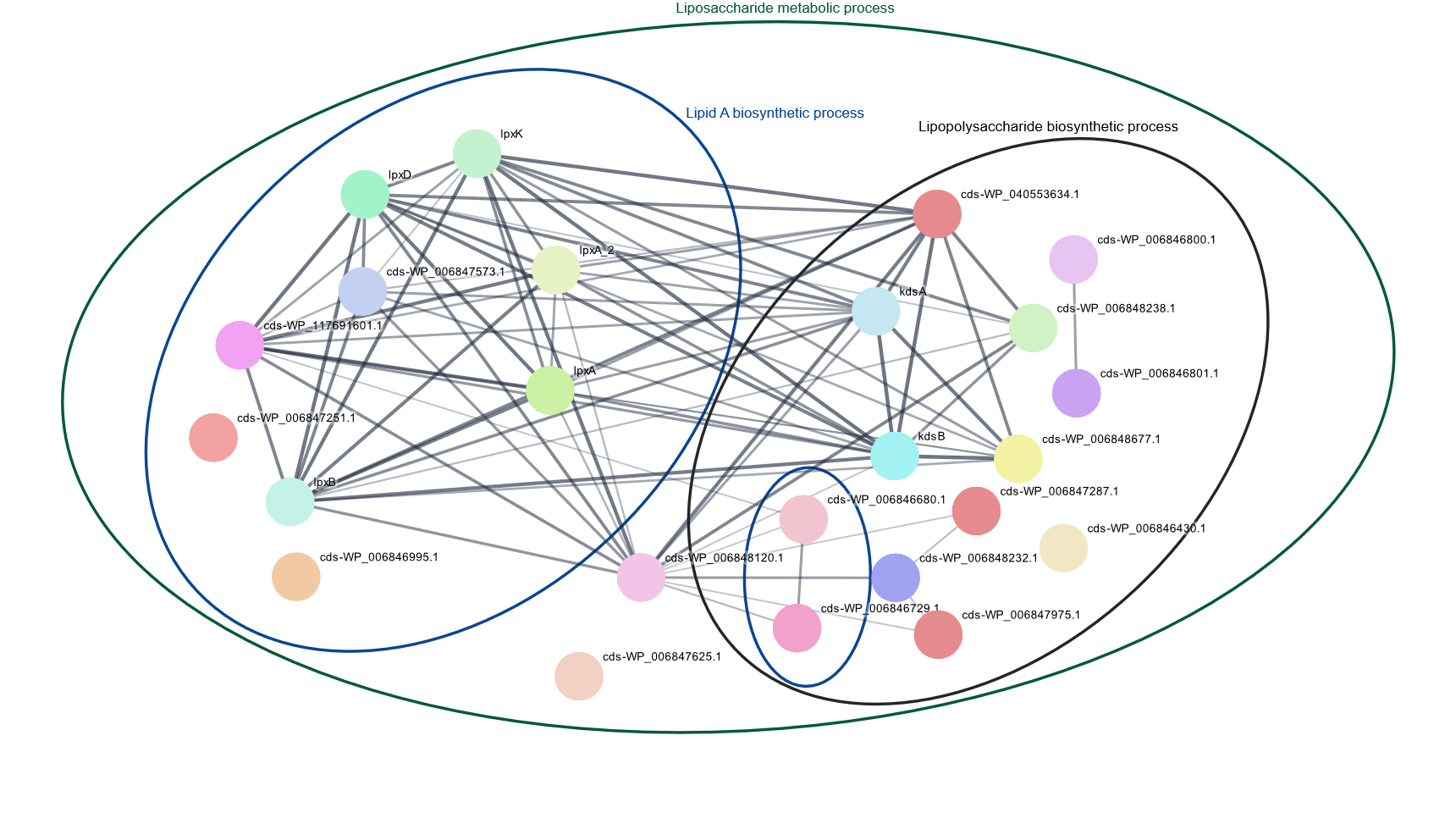
**

**Figure S9.** Functional protein association networks predicted using STRING. Line thickness indicates the strength of data support. Colors represent different Gene Ontology (GO) terms: blue = lipid A biosynthetic process (GO:0009245); black = lipopolysaccharide biosynthetic process (GO:0009103); green = liposaccharide metabolic process (GO:1903509).

| Operon 1229 | Operon 56 |
| --- | --- |
| 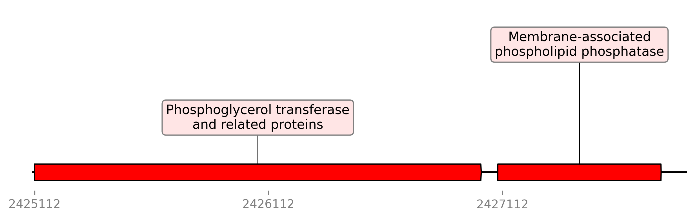 | 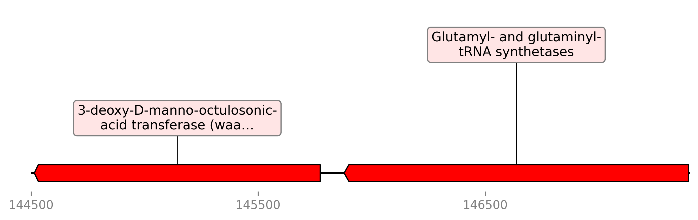 |
| Operon 1030 | Operon 1464 |
| 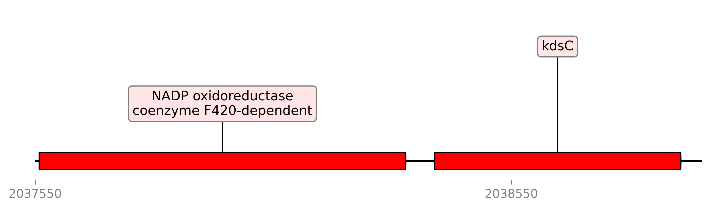 | 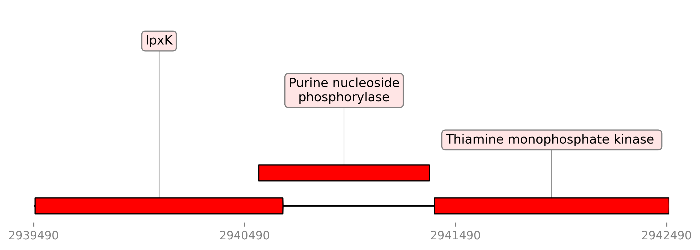 |
| Operon 1222 | Operon 759 |
| 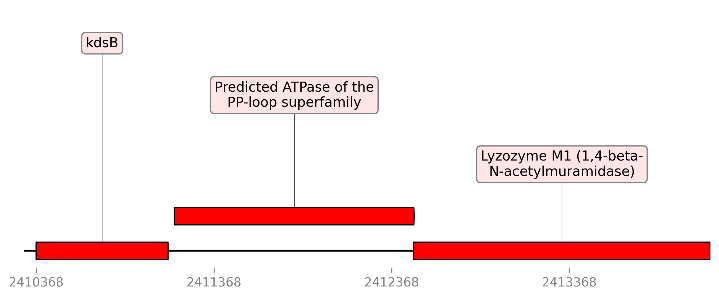 | 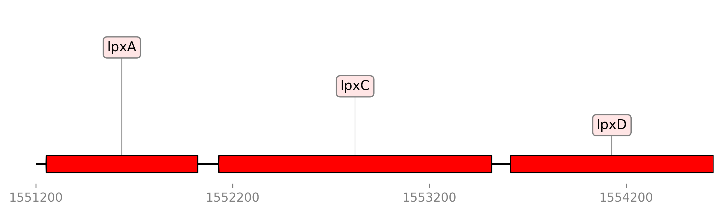 |
| Operon 309 | Operon 1403 |
| 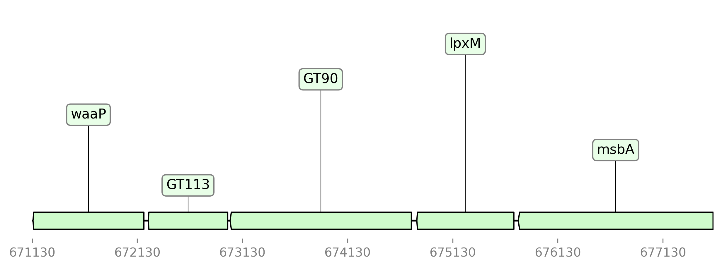 | 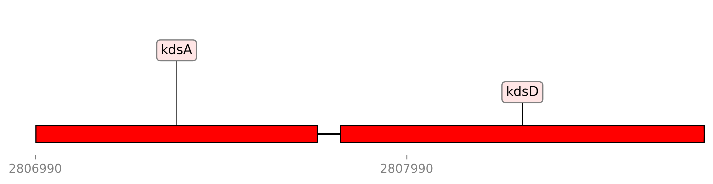 |
| Operon 93 | Operon 712 |
| 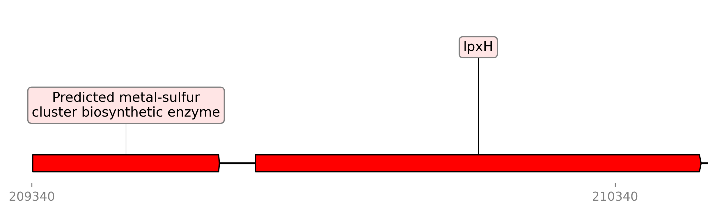 | 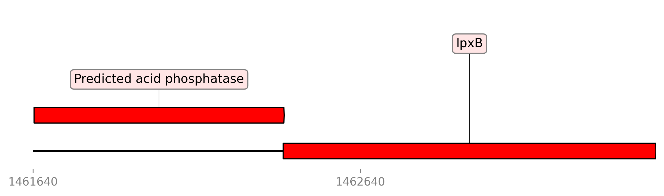 |
| Operon 1564 | Operon 391 |
| 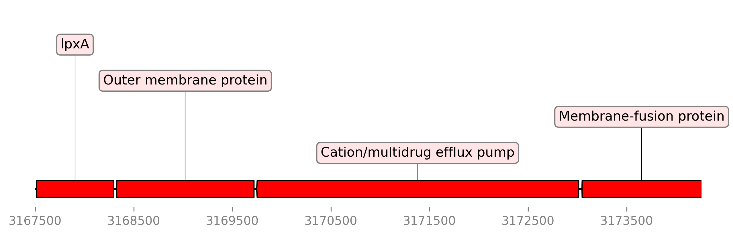 | 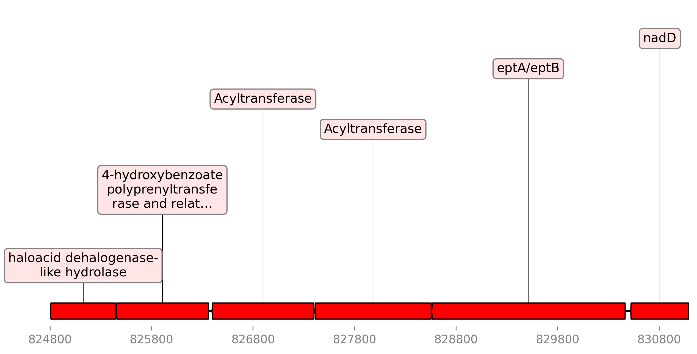 |
| Operon 271 | Operon 790 |
| 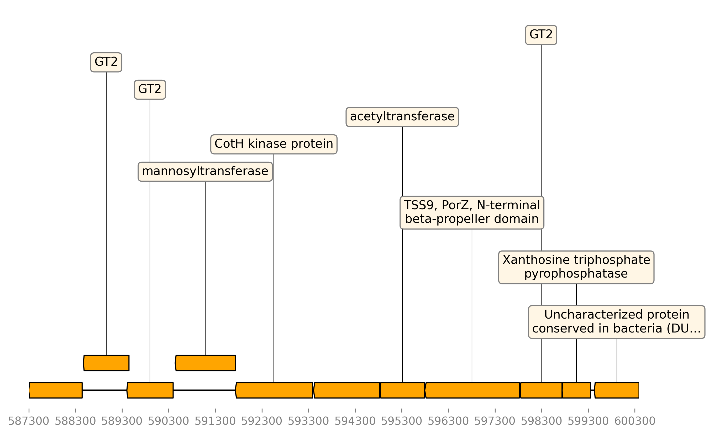 | 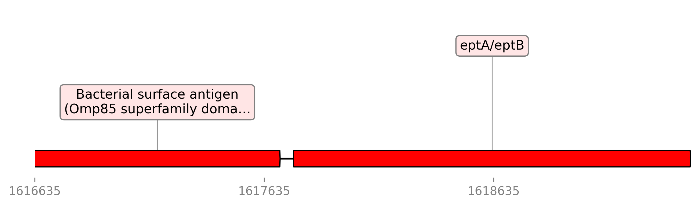 |
| Operon 1410 | Operon 770 |
| 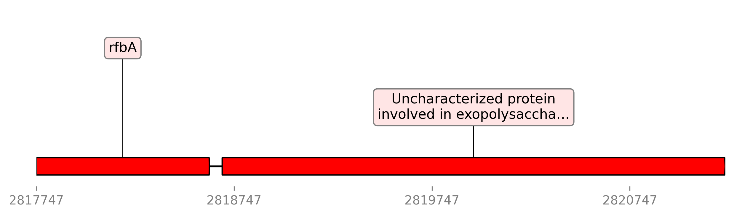 | 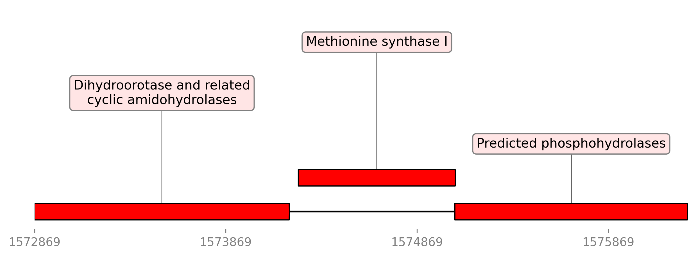 |
| Operon 557 | Operon 320 |
| 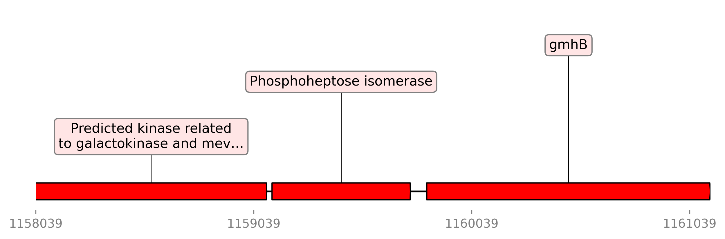 | 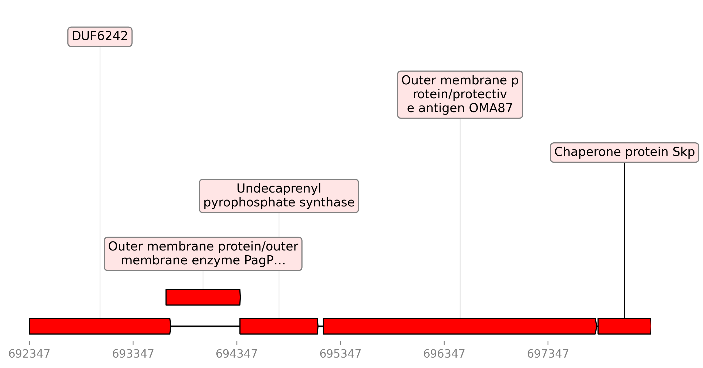 |
| Operon 1250 | Operon 304 |
| 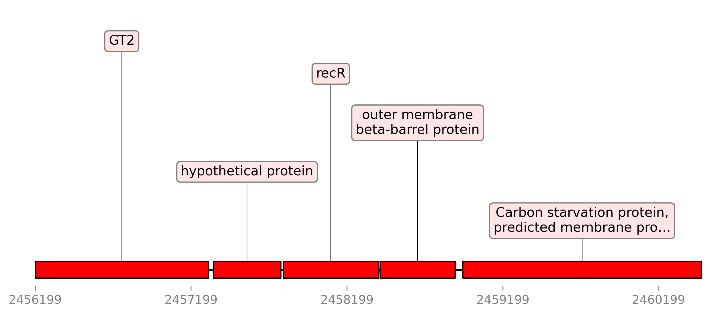 | 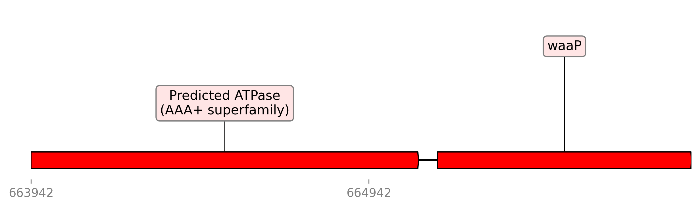 |
| Operon 1204 |  |
| 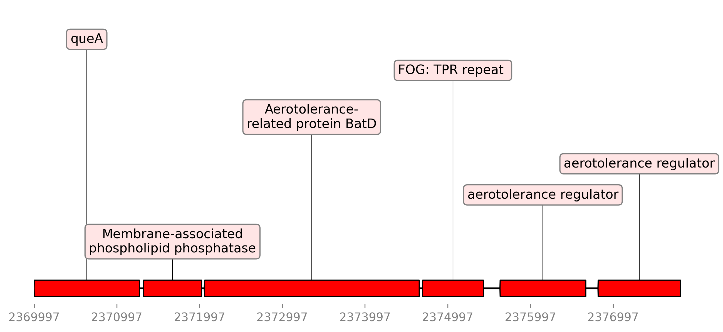 |  |

**Figure S10.** Schematic representation of operons containing one or more genes predicted to be involved in LPS biosynthetic processes. Gene functions were annotated based on their COG classification (predicted using the Operon Mapper web server), while Pfam domains were obtained by submitting the protein sequences to InterProScan. The function of glycosyltransferases was indicated according to their corresponding CAZy family, determined using dbCAN3. Some genes predicted to be involved in LPS biosynthesis are present as single transcriptional units and are not shown here.

**
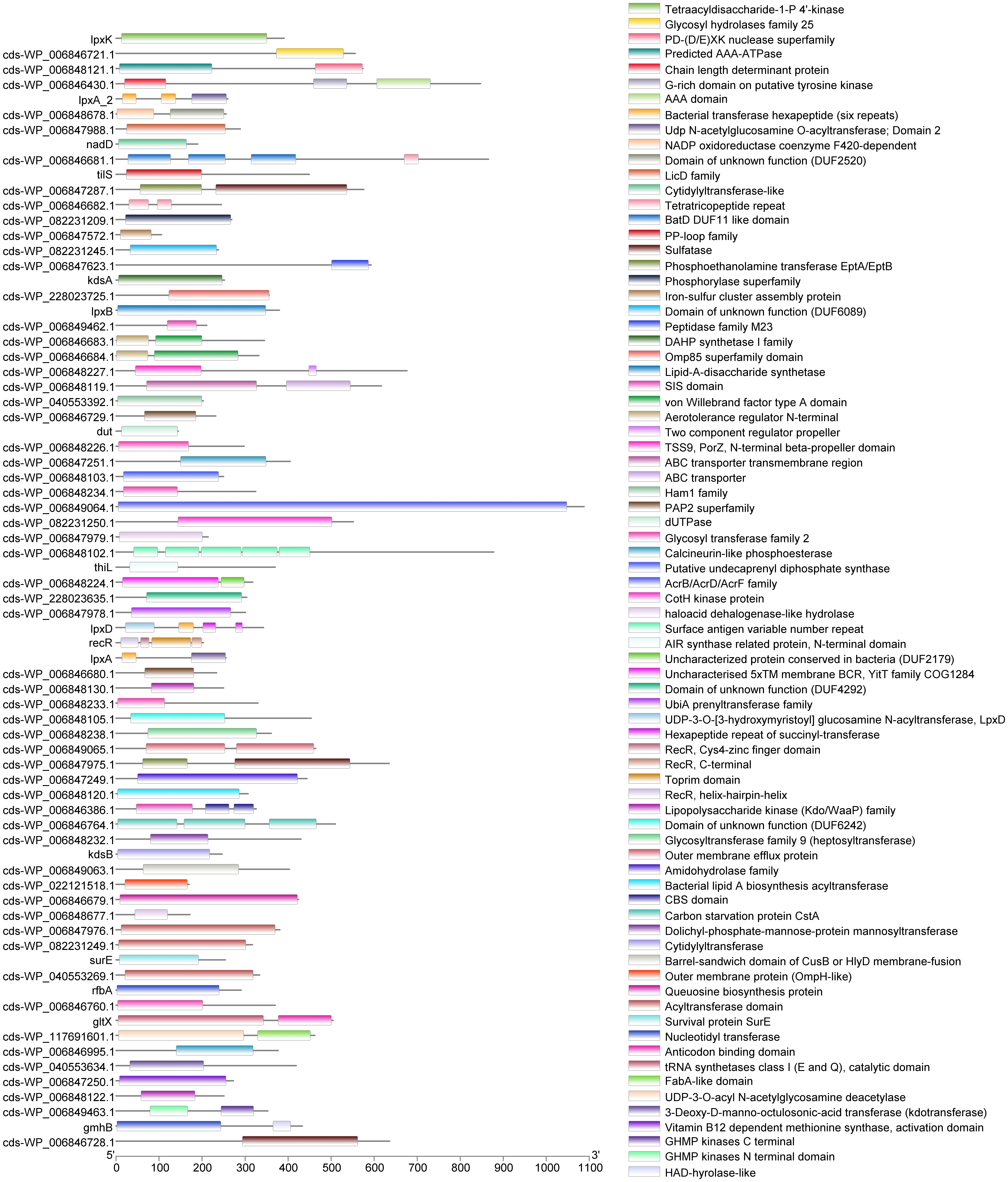
**

**Figure S11.** Predicted Pfam domains for proteins encoded by operons predicted to harbor one or more LPS biosynthesis genes included in the STRING-derived functional networks. Pfam domains were identified by submitting protein sequences to the InterProScan web service. The figure was generated using TB tools-II (version 2.154). Proteins without detectable Pfam domains, lacking significant hits, are not shown.


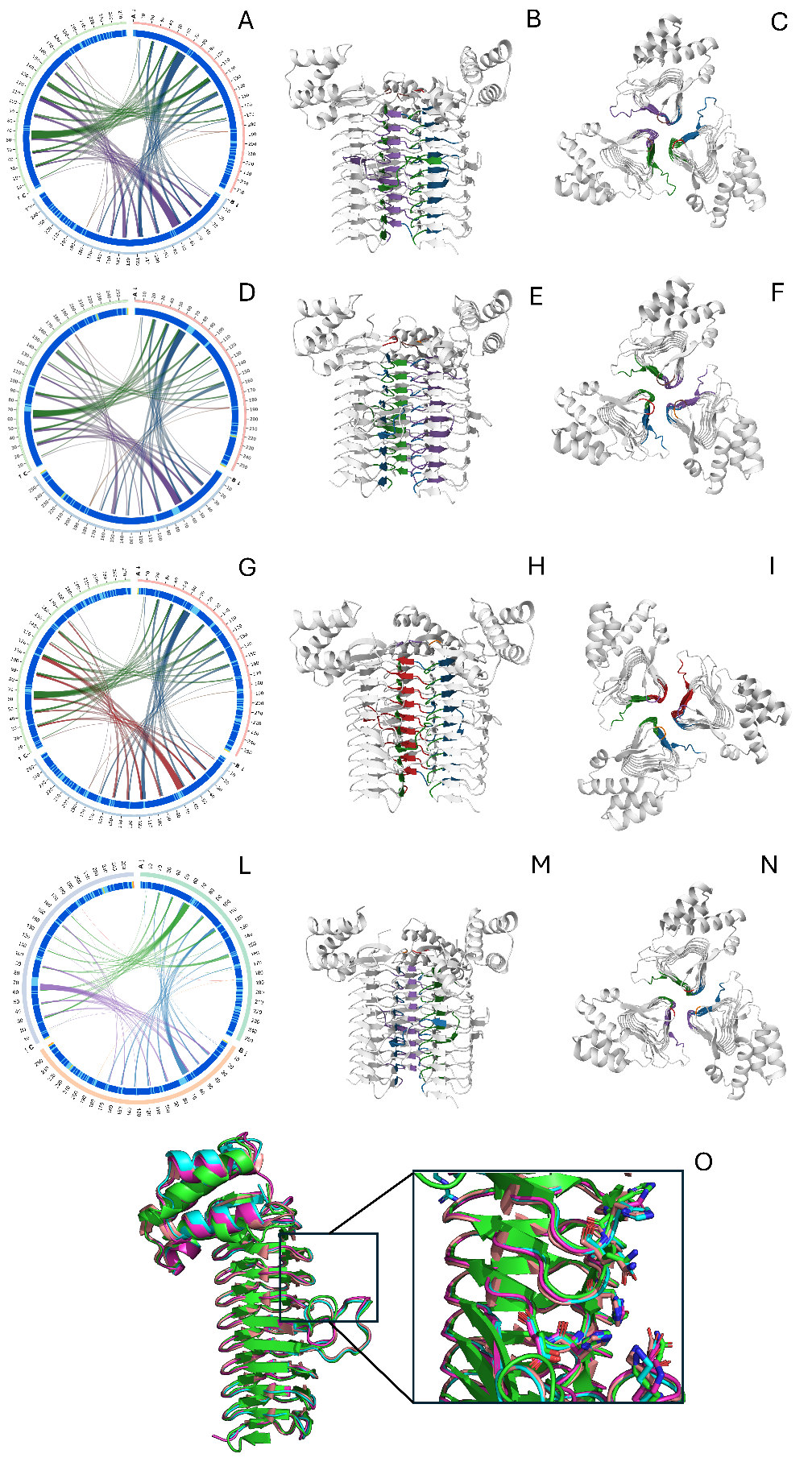
**Figure S12.** *In silico* prediction of the three-dimensional structure of the two LpxA isoforms (lpxA and lpxA_2) in their homotrimeric and hypothetical heterotrimeric conformations. Panels **A**, **D**, **G** and **L** show AlphaBridge diagrams representing predicted interactions between protein subunits; each color corresponds to a distinct pairwise interaction. Panels **A**–**C** refer to the ScLpxA homotrimer, **D**–**F** to the ScLpxA_2 homotrimer, **G**–**I** to the hypothetical heterotrimer composed of two ScLpxA subunits and one ScLpxA_2 subunit and **L**-**N** to the hypothetical heterotrimer composed of two ScLpxA_2 subunits and one ScLpxA subunit. Panel **O** represents the 3D model of ScLpxA_2 (magenta) and ScLpxA (cyan) obtained using AlphaFold3 and the crystallographic structure of BfLpxA (pink; PDB accession: 4R36) superimposed against EcLpxA (green) crystallographic structure (PDB accession: 1lxa) using PyMOL. Superimposition shows evident structural similarity with some variation in flexible loop regions and α-helical domains. The highly conserved catalytic histidine and other residues implied in the formation of the catalytic site are shown.

**Figure S13.** Effects of *S. copri* LPS stimulation on differentiated THP-1 cells. THP1 cells were stimulated for 24 hours with increasing concentrations (0.1-1000 ng/mL) of either *E. coli* LPS or *S. copri* R-LPS to assess: cell viability (**A**), NF-kB activation using Quanti-Blue assay (OD 620nm) (**B**), and TNF-α production by ELISA (**C**). Data are presented as mean ± standard deviation. Statistical comparisons versus unstimulated cells (NS) were analyzed by ordinary one-way ANOVA (^##^*p*-value < 0.01; ^###^*p*-value < 0.001, ^####^*p*-value < 0.0001) (**A**). Statistical differences between *S. copri* R-LPS and *E. coli* LPS at same concentrations, were evaluated using an unpaired *t*-test (***p*-value < 0.01; ****p*-value < 0.001, *****p*-value < 0.0001) (**B,C**).

**
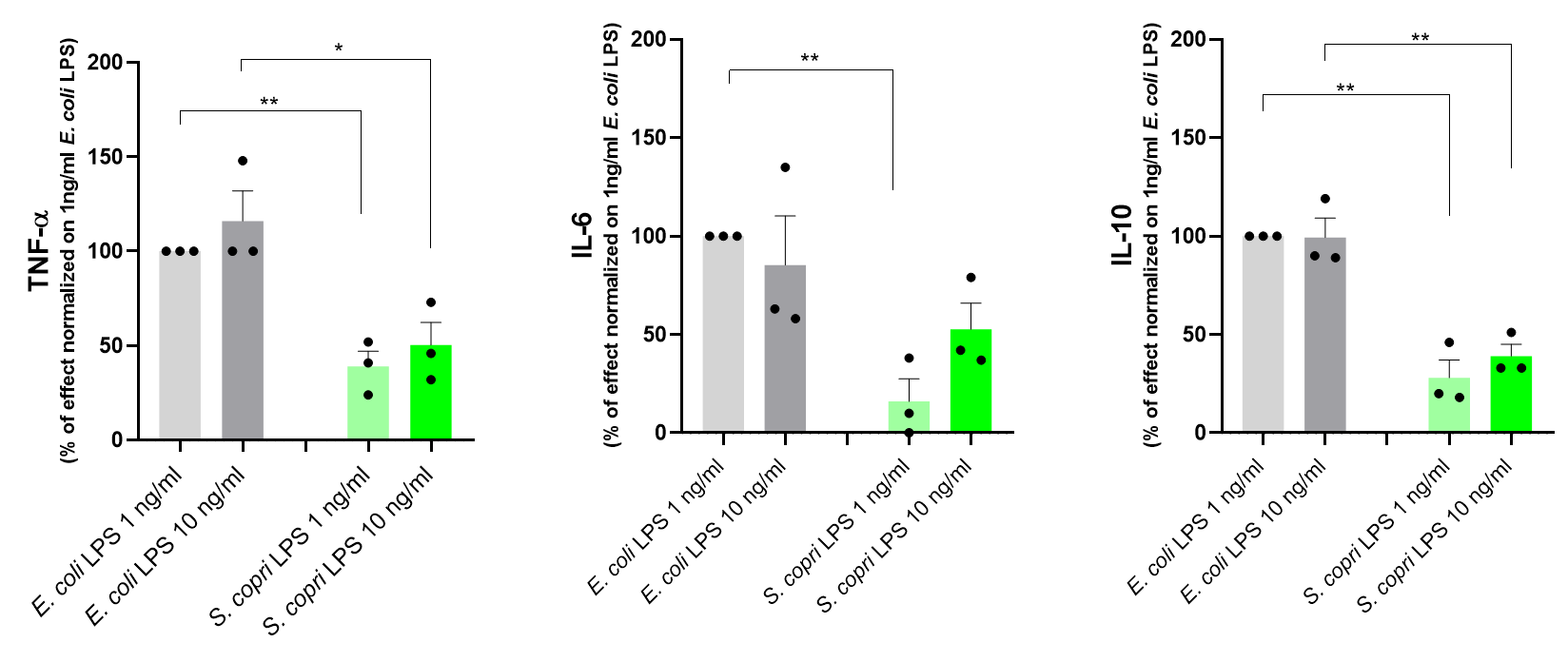
**

**Figure S14.** Cytokine release in PBMCs stimulated with *E. coli* LPS or *S. copri R-*LPS. Human PBMCs (n = 3) were stimulated for 24 hours with the indicated concentrations of LPS. Cytokine levels are shown as a percentage (%) normalized on 1 ng/mL *E. coli* LPS and are presented as mean ± SEM. *t*-test was used to compare the means of each group. **p*-value < 0.05; ***p*-value < 0.01; *E. coli* LPS vs *S. copri* R-LPS.

**Figure S15.** Gating strategy for CyTOF analysis and immune subset identification. Post-acquisition data cleanup was performed according to the Gaussian Discrimination Parameters (GDP) to exclude beads, non-specific signals, Ion cloud fusions (plots 1-6), dead cells (plot 7) and doublets (plots 8-9). From plot 9 were gated the CD45⁺CD3⁻ and CD45⁺CD3⁺ populations (plot CD3⁺/CD3⁻). The CD45^+^CD3^+^ gate was used for the identification of T helper cells (CD4+) and T cytotoxic cells (CD8+) (plot lymphocytes). The CD45⁺CD3⁻ gate was used to define innate immune cell populations (plot innate cells), including CD14⁺ and CD14⁻ subsets. From the CD14⁺ gate, the different monocytes subsets were identified CL: classical CD14⁺CD16^-^; INT: intermediate CD14^++^CD16^+^; NC: non classical CD14^+^CD16^+^ monocytes (plot monocytes). The CD14⁻ compartment allowed discrimination of HLA-DR⁺CD11c⁺ dendritic cells (plot dendritic cells) and CD56⁺CD16⁺ natural killer cells (plot NK).

**Figure S16.** PBMCs immunophenotyping. (**A**) UMAP graphs representing the main PBMCs populations obtained with CyTOF analysis and clustered according to table S6. Analysis was conducted on live cells singlets after quality control and marker selection showed in gating strategy showed in figure S16. Graphs represent the PBMCs untreated control (plot a), treated with *S. copri* R-LPS 0.1 ng/ml (plot b) and with *E. coli* LPS 0.1 ng/mL (plot c). (**B**) Graphic representation of the whole PBMCs subpopulations identified with CyTOF analysis: (a) CD3+/CD3- ratio; (b) T helper, (c) T cytotoxic, (d) natural killer, (e) dendritic cells, (f) monocytes. Mean +/- standard deviation of 6 samples is shown. Statistical analysis was performed by Friedman test with Dunn’s post hoc correction. ***p*-value< 0.01.**Supporting note 1**

**Methods**

**Bacterial strain, growth conditions and cell preparation**

*Segatella copri* DSM 18205 was obtained from the DSMZ, German Collection of Microorganisms and Cell Cultures GmbH, Braunschweig, Germany. It was grown in yeast casitone fatty acid medium with glucose, starch, cellobiose (YCFAGSC)^[50]^ with the following modifications: the short-chain fatty acid mix was omitted, yeast extract was increased to 0.5% final concentration and the final hemin concentration was reduced to 1 mg/ml. To achieve anaerobic conditions, the medium was boiled before and after adding cysteine (0.1% final concentration) as a reducing agent and flushed with carbon dioxide. The pH was set to 7.5 when the medium reached approximately 65⁰C to achieve a final medium pH of approximately 6.5 after autoclaving. Cultures were grown static anaerobically (under carbon dioxide) at 37⁰C overnight. Pre-cultures were grown either in Hungate tubes (10 ml medium) or in 25 ml Wheaton bottles, main cultures were grown after inoculation with 1% (v/v) preculture, either in 100 ml Wheaton bottles, or in a 1 l Duran bottle (800 ml medium) with an anaerobic septum and lid. Cells were harvested by centrifugation in sterile 250 ml Sorvall Lynx bottles (Thermo Fisher Scientific, Paisley, UK) for 15 min at 30239 g (14000 rpm), 4⁰C, in a Sorvall Lynx centrifuge (Thermo Fisher Scientific, Paisley, UK). The supernatant was discarded and the pellet was resuspended in 40 ml deionized sterile water, transferred to 50 ml Falcon tubes and centrifuged for 20 min at 6000 x g, 4⁰C, in Jouan centrifuge MR 18 22 (DJB Labcare Ltd, Newport Pagnell, UK). The supernatant was discarded and the pellet was freeze-dried for 3-4 days in an Edwards Modulyo Freeze Dryer (Edwards Ltd, Burgess Hill, UK).

**LPS isolation and purification**

Lyophilized *Segatella copri* cells (2 g) were extracted using the phenol/chloroform/light petroleum extraction protocol.^[51]^ The extracted material was further purified through multiple steps of ultracentrifugation (100,000 × *g*, 24 h, 4 °C) and gel-filtration chromatography on a Sephacryl High Resolution S-300 (GE-Healthcare, Little Chalfont, UK) column eluted with 50 mM ammonium bicarbonate.^[19]^ Then, it was subjected to sequential organic solvent washes using chloroform/methanol (1:2, v/v) and chloroform/methanol/water (3:2:0.25, v/v), commonly used for phospholipid removal. After complete solvent evaporation, the R-LPS was further processed using the lipoprotein-removal procedure described by Hirschfeld *et al*.^[52]^ The purity of the R-LPS preparation was confirmed by SDS-PAGE and silver staining, with additional assessment for protein or lipoprotein contamination using Coomassie Brilliant Blue staining on parallel gels. Additionally, protein content was quantified using the Pierce Micro BCA Protein Assay (Thermo Scientific, #23235) at a concentration 10000 times higher than that used in cellular assays. To reduce lipid interference, the assay was executed in the presence of 2% SDS. The absorbance was measured at 562 nm using TECAN Infinite M Plex spectrophotometer (Tecan, Grödig,Austria)

**Chemical analyses**

Monosaccharides were identified as acetylated *O*-methylglycoside (AMG) derivatives via GC-MS inspection following methanolysis with 1.25 M HCl in methanol at 85 °C for 16 hours, and subsequent acetylation using acetic anhydride in pyridine (85 °C, 30 min).^[20]^ To determine the absolute configuration of the sugars, *O*-octylglycoside derivatives (OGA) were prepared and analyzed as previously described.^[53]^ Sugar linkage analysis was conducted by converting monosaccharide residues into partially methylated alditol acetates (PMAAs), followed by GC-MS analysis. Briefly, dried LPS aliquot was suspended in DMSO in the presence of powdered NaOH and subjected to alternating stirring and sonication for 2 hours at room temperature before methylation with iodomethane (room temperature for 16h). Then, to transform the methyl ester functions of acidic sugars (such as Kdo) in a hydroxymethyl group with two deuterium atoms, the sample was treated with sodium tetradeuteroborate (NaBD_4_). Following, a hydrolysis with trifluoroacetic acid (2 M, 100 °C, 4 h), carbonyl-reduction by employment again of NaBD_4_, and acetylation with acetic anhydride in pyridine, were performed. The fatty acid profile was assessed by treating the R-LPS sample with 4 M HCl (100 °C, 4 h) followed by a treatment with 5 M NaOH (100 °C, 30 min). After adjusting the pH to ~3, fatty acids were extracted in chloroform, methylated with diazomethane, and analyzed via GC-MS. Ester-linked fatty acids were selectively released through treatment with 0.5 M NaOH in a 1:1 methanol-water mixture at 85 °C for 2 hours. After acidification and extraction in chloroform, they were also methylated and analyzed. Fatty acids were also analyzed as methyl ester derivatives produced during the AMG preparation and compared to those obtained through the above procedures. In addition, an independent methanolysis (1.25 M HCl in methanol, 85 °C, 16 h) was also performed to generate fatty acid methyl esters, which were extracted in hexane and injected into GC-MS. To define the absolute configuration of hydroxylated fatty acids, 3-hydroxy fatty acids were liberated via alkaline hydrolysis (4 M NaOH, 100 °C, 5 h), converted into their 3-methoxy acid L-phenylethylamide derivatives, and analyzed by GC-MS.^[54]^ By comparing retention times with those of standard L-phenylethylamides, the *R*-configuration of all 3-hydroxy fatty acids present in *S. copri* LPS was confirmed. All the above derivatives were inspected on Agilent Technologies Gas Chromatograph 7820A equipped with a mass selective detector 5977B and an HP-5 capillary column (Agilent, Milan, Italy 30 m × 0.25 mm i.d., flow rate 1 mL/min, He as carrier gas). The temperature program used to inspect AMG and OGA was: 140 °C for 3 min, then 140 → 240 °C at 3 °C/min. The temperature program for PMAA was: 90 °C for 1 min, then 90 → 140 °C at 25 °C/min, then 140 → 200 °C at 5 °C/min, then 200 → 280 °C at 10 °C/min, and finally 280 °C for 10 min. To analyze fatty acid temperature program used was: 150 °C for 5 min, 150 to 280 °C at 3 °C/min, and 280 °C for 5 min.

**Isolation of the core OS and lipid A**

An aliquot of purified R-LPS was subjected to *O*-deacylation by treatment with anhydrous hydrazine (2 mL) under stirring at 37 °C for 90 minutes. After cooling, the reaction mixture was added to ice-cold acetone (20 mL). The resulting precipitate was collected by centrifugation (4000 × g, 30 min), washed thoroughly with cold acetone, dried, redissolved in water, and lyophilized. The resulting *O*-deacylated product was then subjected to *N*-deacylation using 4 M KOH (120° C, 16 h). To eliminate salts, the sample was passed through a Sephadex G-10 gel-filtration column (50 × 1.5 cm, Pharmacia). Final purification of the fully deacylated product was achieved by size-exclusion chromatography on a Toyopearl TSK HW-40 column (Tosoh Bioscience) eluted with 50 mM ammonium bicarbonate. The sample was then analyzed via 1D and 2D NMR. In parallel, another aliquot of R-LPS was hydrolyzed in acetate buffer (pH 4.4) at 100 °C for 2 hours under constant magnetic stirring. After hydrolysis, a mixture of methanol and chloroform was added to the reaction mixture to achieve a final volume ratio of CH₃OH/CHCl₃/hydrolysate 2:2:1.8 (v/v/v). The resulting suspension was vigorously mixed and centrifuged (8800 × g for 20 minutes at 4 °C). The chloroform layer, containing the lipid A fraction, was separated and washed with the aqueous layer of a freshly prepared Bligh and Dyer mixture (CH_3_Cl/CH_3_OH/H_2_O, 2:2:1.8, v/v/v). ^[55]^ Organic phases were then combined, dried, and analyzed by MALDI-TOF mass spectrometry to characterize the lipid A moiety.

**NMR Spectroscopy**

1D and 2D NMR spectra were recorded in D_2_O at 298 K at pD = 7 with a Bruker 600 AVANCE NEO instrument equipped with a cryoprobe. The spectra were calibrated with internal acetone (*δ*_H_ = 2.225 ppm; *δ*_C_ = 31.45 ppm). ^31^P NMR experiments were carried out with a Bruker DRX-400 spectrometer; aqueous 85 % phosphoric acid was used as external reference (*δ* = 0.00 ppm). Double-quantum-filtered phase sensitive correlation spectroscopy (DQF-COSY) experiments were recorded by using data sets of 4096 × 512 points. Total correlation spectroscopy (TOCSY) experiments were carried out with spinlock times of 100 ms using data sets (t1 × t2) of 4096 × 256 points. Rotating frame Overhauser enhancement spectroscopy (ROESY) was performed using data sets (t1 × t2) of 4096 × 256 points and mixing times between 100 and 300 ms, acquiring 16 scans. The data matrix in all the homonuclear experiments was zero-filled in both dimensions to give a matrix of 4K × 2K points and was resolution-enhanced in both dimensions by a cosine-bell function before Fourier transformation. Coupling constants were attained from 2D phase-sensitive DQF-COSY.^[56]^ Heteronuclear single quantum coherence (^1^H,^13^C HSQC) and heteronuclear multiple bond correlation (^1^H,^13^C HMBC) experiments were carried out in 1H-detection mode by single-quantum coherence with proton decoupling in the ^13^C domain using data sets of 2048 × 256 points. ^1^H,^13^C HSQC was performed using sensitivity improvement and in the phase-sensitive mode using Echo/Antiecho gradient selection, with multiplicity editing during selection step. ^1^H,^13^C HMBC was optimized on long range coupling constants, with low-pass J-filter to suppress one-bond correlations, using gradient pulses for selection. A 60 ms delay was adopted for the evolution of long-range correlations. ^1^H,^13^C HMBC was optimized for a 6 Hz coupling constant, and ^1^H,^31^P HSQC was optimized for an 8 Hz coupling constant. The data matrix in all the heteronuclear experiments was extended to 2048 × 1024 points by using forward linear prediction extrapolation.^[21]^

**ESI Mass spectrometry**

R-LPS was dissolved in a 1:1 (vol/vol) mixture of 2-propanol and water and directly infused by syringe pump (10 μL min^−1^, at an estimated concentration of 20 μg mL^−1^) into the source of a Synapt XS mass spectrometer (Waters, Manchester, UK), equipped with a traveling wave IMS (TWIMS) and a 8 kDa quadrupole operating in negative polarity, electrospray ionization (ESI) and Resolution mode. The analyses were operated with a capillary potential of -2.1 kV, source temperature of 120 °C, sampling cone at 20.0 V, source offset at 10 V, source gas (N_2_) flow at 0.0 mL min^−1^, desolvation temperature of 350 °C, cone gas flow at 40 L hr^−1^, desolvation gas flow at 300 L hr^−1^, and nebulizer gas pressure at 3.0 bar. Nitrogen was supplied by a PLINIUS N45-1 nitrogen generator from CLAIND and was used as desolvation and cone gas. MS/MS experiments were performed using ultra-pure argon (SOL SpA) as collision gas in the trap cell, with a collision energy ramp ranging from 20V to 120 V depending on the selected precursor ion. A 2 µg µL^-1^ solution of NaI in 1:1 (vol/vol) 2-propanol/H_2_O was used for the calibration from 50 to 4000 Da. Data acquisition was performed with MassLynx^TM^ software (V4.2) while the reported MS and MS/MS spectra images were generated using Waters Connect software. A reference solution consisting of 100 pg µL^-1^ Leucine Enkephalin dissolved in 1:1:0.1 (vol/vol/vol) mixture of water/acetonitrile/formic acid 0.1% was used as lock mass during sample acquisition with a lock spray capillary of -2.6 kV and a scan time of 0.5 sec. A lock mass correction and a background subtraction were applied by MassLynx after the acquisition, using the average of 3 scans on the reference channel. Traveling wave ion mobility separation (TWIMS) analysis were performed using an IMS wave velocity of 850 m s^−1^ and a wave height of 40.0 V. The ESI source was operated in negative polarity and resolution mode with a capillary potential of -2.1 kV, source temperature of 120 °C, sampling cone at 60.0 V, source offset at 0 V, source gas (N2) flow at 0.0 mL min^−1^, desolvation temperature of 300 °C, cone gas flow at 40 L hr^−1^, desolvation gas flow at 350 L hr^−1^, and nebulizer gas pressure at 3.0 bar. For the core OS, glycosidic and cross-ring fragments interpretation was complemented with GlycoWork bench-assisted annotation.^[57,58]^

**MALDI-TOF Mass spectrometry**

MALDI-TOF mass spectrometry analyses were performed using an ABSCIEX TOF/TOF™ 5800 instrument (Applied Biosystems) equipped with an Nd:YAG laser (λ = 349 nm, 3 ns pulse width, up to 1000 Hz repetition rate) and delayed extraction capabilities. The matrix for lipid A analysis was prepared by dissolving trihydroxyacetophenone (THAP) in a methanol/0.1% trifluoracetic acid/acetonitrile mixture (7:2:1, v/v/v) at a concentration of 75 mg/mL, while lipid A fraction was dissolved in chloroform/methanol (1:1, v/v). For direct analysis of bacterial pellets, 2,5-dihydroxybenzoic acid (DHB) at 10 mg/mL in chloroform/methanol (9:1, v/v) was used as the matrix.^[59]^ A volume of 0.5 μL of the sample was mixed with 0.5 μL of matrix solution and spotted onto the MALDI plate, then dried under vacuum at room temperature. Different matrix-to-sample ratios were tested, with a 1:1 ratio yielding optimal signal-to-noise performance. Lipid A derived from *E. coli* O127:B8 LPS, prepared in-house by mild acid hydrolysis, served as a reference standard. All analyses were carried out in technical triplicates and repeated on three independent lipid A preparations derived from separate mild acid hydrolysis reactions of R-LPS. In addition, an aliquot of intact R-LPS was resuspended in 5 mM EDTA, gently agitated, and briefly treated in an ultrasonic bath to aid dispersion. Desalting was performed using cation-exchange resin (Dowex 50X, NH_4_^+^ form), after which the sample was mixed in equal volume with a matrix solution consisting of 2,5-dihydroxybenzoic acid (DHB) dissolved in 0.1% citric acid. A 1 μL aliquot of this LPS/matrix mixture was then applied onto a stainless-steel MALDI target plate. Mass spectra of the intact LPS, isolated lipid A and bacterial pellet were acquired in negative ion mode using the reflectron mode. Each MS spectrum was averaged from 3000 laser shots, while MS/MS spectra were obtained by accumulating 5000–7000 shots. Acquisition was performed ensuring random yet uniform sampling across the sample spot.

**Bioinformatic analysis**

Annotations of the *S. copri* DSM18205 genome available in the National Center for Biotechnology Information (NCBI) database (RefSeq accession: GCF_020735445.1) were used for this analysis (https://www.ncbi.nlm.nih.gov/). Using the Operon-mapper web server,^[60]^ we determined the possible operons present in the genome together with the predicted functions and COG for each gene. The operons containing genes with predicted LPS biosynthesis associated COG and/or function presents in the KEGG database were considered associated with LPS biosynthesis. Importance was given to the genes present in the KEGG pathway map00540. Once obtained also all the predicted protein sequences for *S. copri* genome, we uploaded them to STRING (<https://string-db.org>) to generate an interaction network and a prediction of the biological function. For the sake of these analysis, we only considered the networks based on Gene Ontology terms and KEGG pathways. The interaction networks were visualized in Cytoscape software (version 3.10.3 [http://cytoscape.org]). The protein sequences predicted to be part of LPS biosynthesis network or genetic region were further analysed using InterProScan (https://www.ebi.ac.uk/interpro) to determine the Pfam (hits with e-values higher than 1e-05 were considered unreliable) and retrieve other potentially useful information regarding the predicted function.^[61]^ The Pfam were visualized using TB tools-II (version 2.154).^[62]^ For glycosyltransferases, the CAZy family was determined using dbCAN3.^[63]^ The operons most likely involved in the R-LPS biosynthesis were visualized using a custom script based on the DNA Features Viewer library.^[64]^ To predict the 3D structure of the protein complex of interest AlphaFoldServer^[30]^ was employed. The aminoacidic sequences, in FASTA format, were processed through the ColabFold implementation of AlphaFold 3.0,^[30]^ that makes possible the modelling of protein-protein complexes as well as individual protein structures. The confidence of the model was assessed through the predicted aligned error (PAE) and confidence scores (pLDDT and interface pTM scores). The AlphaFold 3.0 models obtained were used as input for AphaBridge,^[31]^ a computational tool for protein-protein docking refinement, which evaluate residue-residue interactions in the predicted binding interface identifying residue pairs with high likelihood of producing stable contact. Structural superimposition of one of the predicted *S. copri* LpxA model onto the *E. coli* LpxA monomer (PDB ID: 1lxa) was performed using PyMOL. Structural superposition of the two predicted *S. copri* LpxA models against crystallographic structures of homologous proteins from the RCSB PDB^[65]^ was performed again using PyMOL.^[66]^

**Cell Culture**

HEK-Blue hTLR4, HEK-Blue hTLR2, HEK-Blue TLR2/1, HEK-Blue TLR2/6 and THP1-Blue NF-κB cell lines were provided by Invivogen (Invivogen, Toulouse, France). HEK-Blue hTLR4, HEK-Blue hTLR2, HEK-Blue TLR2/1 and HEK-Blue TLR2/6 cells were cultured in Dulbecco’s Modified Eagle’s Medium (DMEM) 4 g/L glucose supplemented with 10% heat-inactivated fetal bovine serum (FBS), 1% penicillin/streptomycin (Pen/Strep), 2 mM L-glutamine and 100 μg/ml Normocin. To ensure plasmid selection, HEK-Blue selection was added to HEK-Blue cell culture medium according to the manufacturer's instructions. THP1-Blue NF-κB cells were grown in RPMI 1640 medium supplemented with 10% heat-inactivated FBS, 1% Pen/Strep, 2 mM L-glutamine, 25 mM HEPES, 100 μg/ml Normocin and 10 µg/mL Blasticidin. All cell lines were maintained at 37°C in a humidified atmosphere containing 5% CO_2_.Cell culture reagents including FBS, Pen/Strep, DMEM, RPMI 1640, L-glutamine and HEPES were purchased from Thermofisher (Gibco, Thermo Fisher Scientific), while Normocin, Blasticidin and HEK-Blue selection were purchased from Invivogen (Invivogen, Toulouse, France).

**LPS stimulation of HEK and THP-1 cells**

HEK-Blue hTLR4, HEK-Blue hTLR2, HEK-Blue TLR2/1 and HEK-Blue TLR2/6 cells were seeded into a 96-well plate (3 x 10^4^/well). After an overnight attachment, HEK-Blue cells were stimulated for 18 hours with various concentrations (1-10-100 ng/mL for HEK-Blue hTRL4 and HEK-Blue hTLR2 cells; 100 ng/mL for HEK-Blue TLR2/1 and HEK-Blue TLR2/6 cells) of *E. coli* O111:B4 LPS (Invivogen, Toulouse, France) or *S. copri* R-LPS. NF-kB activation was measured by Quanti blue assay (Invivogen, Toulouse, France). HEK-Blue hTLR2 and HEK-Blue TLR2/1 cells were also treated with 500 ng/mL Pam3CSK4 (Invivogen, Toulouse, France), a positive control of TLR2 activation. Instead, FSL-1 (Invivogen, Toulouse, France), a TLR2/6 agonist, at a concentration of 100 ng/mL was used as a positive control of TLR2 heterodimerization with TLR6 receptor. THP1-Blue NF-κB cells were seeded into a 96-well plate (6 x 10^4^ cells/well) and differentiated following 24-hour treatment with 5 ng/mL phorbol 12-myristate 13-acetate (PMA) (Thermoscientific, Kandel, Germany) and 72-hour resting in PMA-free medium. After differentiation, THP1 cells were treated with various concentrations (0.1-1-10-100-1000 ng/mL) of either *E. coli* LPS or *S. copri* R*-*LPS for 24 hours. The secreted embryonic alkaline phosphatase (SEAP) levels from HEK-Blue hTLR4, HEK-Blue hTLR2, HEK-Blue TLR2/1, HEK-Blue TLR2/6 and THP1-Blue NF-κB cell supernatants were evaluated to establish NF-kB activation following LPSs stimulation by Quanti-Blue assay. For this assay, the absorbance at 620 nm was measured by using TECAN Infinite M Plex spectrophotometer (Tecan, Grödig, Austria). For competition assay, HEK-Blue hTLR4 cells were primed with either *Rhodobacter sphaeroides* (RS) LPS (#tlrl-prslps, Invivogen, Toulouse, France) or *S. copri* R-LPS (1-10-100 ng/mL) for 4 hours and then stimulated with 10 ng/mL *E. coli* LPS for 16 hours. The absorbance results from Quanti-blue assay were expressed as a percentage versus 10 ng/mL *E. coli* LPS values considered 100%.

**3- (4,5-dimethylthiazol-2-yl)-2,5-diphenyltetrazolium bromide (MTT) assay**

Cell viability was assessed by MTT assay on PMA-differentiated THP1 cells. MTT (#BID2165, Apollo Scientific) was dissolved in PBS to obtain 5 mg/mL stock solution and filtered through 0.22 µm pore-size filters. Following 24-hour stimulation with either *E. coli* LPS or *S. copri* R-LPS, culture supernatants were removed, and cells were incubated with medium containing 0.5 mg/mL MTT for 3 hours at 37°C. Then, the medium was removed, and the resulting formazan crystals were solubilized using dimethyl sulfoxide (DMSO). Absorbance was measured at 570 nm with background subtraction at 630 nm using TECAN Infinite M Plex spectrophotometer (Tecan, Grödig, Austria). Cell viability data were normalized towards unstimulated control cells.

**Enzyme-linked immunosorbent assay (ELISA) assay**

TNF-α levels in the supernatant of PMA-differentiated THP1 cells stimulated for 24 hours with either *E. coli* LPS or *S. copri* R-LPS were assessed by Duoset ELISA kits following the manufacturer's instructions (R&D system, Bio-Techne, Minneapolis, USA). Optical density at 450 nm with background subtraction at 570 nm was determined by using TECAN Infinite M Plex spectrophotometer (Tecan, Grödig, Austria).

**Peripheral blood mononuclear cells (PBMCs) isolation**

PBMCs were isolated from the peripheral blood of six healthy donors recruited at San Paolo Hospital in Naples (Italy) using density gradient centrifugation with Ficoll-Paque™ PLUS (GE Healthcare, Chicago, IL, USA). No identifying donor information was collected. The study was conducted in accordance with the ethical standards of the institutional review board and the 1964 Declaration of Helsinki. PBMCs were seeded at a concentration of 2 × 10^6^ cells per well in 2 mL of RPMI 1640 medium supplemented with 10% fetal bovine serum (FBS) and 1% penicillin-streptomycin (all from Thermo Fisher Scientific, Waltham, MA, USA), in 6-well plates.

**Cytokines production in PBMCs by LPS**

PBMCs were stimulated with LPS derived from *E. coli* and *S. copri* at concentrations of 1 ng/mL and 10 ng/mL for 24 h. The cellular response was assessed by evaluating the expression levels of key cytokines, including IL-10, IL-6, and TNF-α. Cytokine quantification was performed using the ultrasensitive ELLA-Simple Plex technology (ELLA microfluidic analyzer, Protein Simple; Bio-techne, San José, CA, USA), utilizing a customized kit.

**CyTOF analysis**

PBMCs were stimulated for 1 hour with *S. copri* or *E.* *coli* LPS at final concentrations of 0.01 or 0.1 ng/mL. Then, PBMCs were harvested and washed in 1 mL of ultrapure PBS. Cells were then stained with cisplatin (Standard BioTools, South San Francisco, CA, USA) at a final concentration of 1 µM for 5 minutes at room temperature to exclude dead cells. After incubation, cells were washed with Cell Staining Buffer (CSB; Standard BioTools) and resuspended in 50 µL of CSB. The antibody cocktail was prepared according to the manufacturer’s recommendations using the following metal-conjugated monoclonal antibodies (all from Standard BioTools): CD45-89Y, CD3-141Pr, CD4-144Nd, CD8-168Er, HLA-DR-170Er, TLR4-158Gd, CD16-148Nd, CD14-175Lu, CD11c-147Sm, CD56-149Sm, and CD86-156Gd (Table S6). A volume of 50 µL of antibody mix was added to the cell suspension (final volume 100 µL), and samples were incubated for 30 minutes at room temperature. Following two washes with CSB, cells were fixed in freshly prepared 1.6% methanol-free paraformaldehyde (PFA; Thermo Fisher Scientific, Waltham, MA, USA) for 10 minutes at room temperature. After centrifugation at 800×g for 5 minutes, cells were resuspended in Cell-ID™ Intercalator-Ir (Standard BioTools) diluted in Maxpar® Fix and Perm Buffer (Standard BioTools) to a final concentration of 125 nM and incubated overnight at 4 °C prior to acquisition. On the day of acquisition, cells were washed twice with CSB and twice with Cell Acquisition Solution (CAS; Standard BioTools), according to the manufacturer’s protocol. Finally, cells were resuspended at 1 × 10⁶ cells/mL in CAS supplemented with 0.1× EQ™ Four Element Calibration Beads (Standard BioTools). Samples were acquired using a Helios™ 2 mass cytometer (Standard BioTools) after performing instrument tuning and bead sensitivity calibration. A total of 300,000 events were recorded at an acquisition rate of 250–500 events per second using a medium flow rate. CyTOF data were exported as FCS files after normalization by using CyTOF® Software v7.0.5189 (Standard BioTools, USA), based on EQ™ Four Element Calibration Beads-based passport. Normalized FCS files were imported into the OMIQ platform (Omiq Inc., part of Dotmatics, Boston, MA, USA) for data cleanup using Gaussian Discrimination Parameters (GDP), high-dimensional data analysis, including gating strategies and clustering. Dimensionality reduction was conducted with Uniform Manifold Approximation and Projection (UMAP) using as parameters a minimum distance of 0.4 and 15 nearest neighbors. Population frequencies (%) and median marker expression values were exported from OMIQ for statistical analysis.

**Statistical analysis**

Data are expressed as mean ± standard deviation (SD) unless otherwise indicated. For the ELLA assay, cytokine expression levels are presented as the mean ± standard error of the mean (SEM). Statistical differences were assessed by ordinary one-way ANOVA for experiments performed on HEK cells and for the MTT assay on THP-1 cells. NF-κB activation and TNF-α release in THP-1 cells, as well as cytokine release in PBMCs, were evaluated using unpaired t-tests. For all tests, a *p*-value < 0.05 was considered statistically significant. All statistical analyses were performed using GraphPad Prism software (version9.1.2 GraphPad Software, CA, USA). Immune cells population frequencies obtained with CyTOF were presented as percentage (%) and non-parametric one-way ANOVA test followed by Tukey’s post hoc multiple comparisons test were used for statistics. p-values < 0.05 were considered statistically significant also in this case.

**Supporting note 2.**

**NMR characterization of the core OS of *S. copri* R-LPS**

The ^1^H and the ^1^H,^13^C HSQC spectra of the core OS obtained after full deacylation of the R-LPS (Figure 1A), showed eleven anomeric signals, indicative of eleven spin systems (**A**–**H**, Figure 1; Table S2). Upfield-shifted signals at 2.14/1.93 and 2.14/1.91 ppm were ascribed to the H-3 methylene protons of the 3-deoxy-D-*manno*-oct-ulosonic acid (Kdo) (**K/K’**3, Figure 1; Table S2). All monosaccharide residues were present as pyranose rings, as indicated by their ^13^C NMR chemical shift values and in accordance with compositional analysis. Spin systems **A** and **F**/**F’**, with anomeric proton signals at *δ* 5.52 ppm and *δ* 4.95/4.92 ppm respectively, were assigned to α- and β-glucosamine (D-GlcN) units of the lipid A domain. This assignment was supported by correlations of their H-2 protons with nitrogen-bearing carbon atoms resonating at *δ*_C_ 54.2 and 55.5 ppm (Table S2, Figure 1A). Additional confirmation came from a NOE correlation between H-1 of residue **F**/**F’** and the H-6 protons of residue **A** (Figure S4). Moreover, in the ^31^P,^1^H HSQC spectrum (not shown), a correlation between a phosphate signal at δ 3.61 ppm and the anomeric proton of residue **A** (*δ*_H_ 5.52 ppm) enabled assignment of a phosphate group to this position. The occurrence of two spin systems (**F**/**F’**) for lipid A β-D-GlcN unit was due to the non-stoichiometric phosphorylation of residue **B** (as described below). No evidence of phosphorylation at the O4 position of **F**/**F’** was noticed by NMR analysis, suggesting that such a modification occurs only in minor species, as shown by MS analysis. Spin systems **B**/**B’** (H-1/C-1 at 5.31-5.19/98.5-98.2ppm), **C** (H-1/C-1 at 5.20/100.3 ppm) and **E** (H-1/C-1 at 4.96/102.0 ppm) were attributed to α-mannose residues (α-D-Man), based on the small scalar coupling constants ^3^*J*_H1,H2_ and ^3^*J*_H2,H3_ (< 3 Hz), indicative of an equatorial orientation of the H-2 proton, and by the *intra*-residue NOE of H-1 only with H-2. The downfield shift at *δ* 3.77 ppm for H-4 of α-D-Man **B’** value and the cross-peaks in the ^31^P,^1^H HSQC spectrum (not shown) with signals at δ 4.10 ppm for H-4 **B’** provided clear evidence of *O*-phosphorylation at this position. However, as mentioned above, its phosphorylation was not stoichiometric which generated spin system **B** and resulted in the splitting of some other NMR signals including **B’**, **G’**, **K’** and **F’**. Spin systems **D** (H-1/C-1 at 5.02/98.0 ppm) and **G/G’** (H-1/C-1 at 4.70-4.42/102.3-102.0ppm) were attributed to *gluco*-configured units due to their high ^3^*J*_H,H_ ring proton values and the chemical shift values, in agreement with *gluco*-configuration of pyranose rings.^[21]^ Anomeric β-configuration of **G/G’** was deduced by the observation of the *intra*-residue NOE contact of H-1 with H-3 and H-5, and the ^3^*J*_H-1,H-2_ value (~8 Hz); whereas the anomeric α-configuration of **D** was assessed by the ^3^*J*_H-1,H-2_ coupling constant values and the NOE correlation between H-1 and H-2. As for the Kdo unit (**K/K’**), this was identified starting from its characteristic diastereotopic methylene proton signals, while its α-anomeric configuration was inferred from the chemical shift of H-3 and the coupling constants ^3^*J*_H7,H8a_ and ^3^*J*_H7,H8b_.^[67]^ Moreover, the Kdo was found to bear a 2-aminoethyl phosphate (*P*EtN) unit at its O-4 position, as proven by the correlation of H-4 **K/K’** with a signal at -0.75 ppm in the ^31^P,^1^H HSQC spectrum, in turn correlating with methylene proton resonances at *δ*_H_ 3.95 and 3.26 ppm (Figure 1A).

Down-field shifted carbon signals were indicative of glycosylation at position O-6 of **A**, **F**/**F’**, and **H**, at O-2 of **C** and **D**, at O-2 and O-6 of **B**/**B’**, and at O-5 of **K/K’**, whereas **E** and **G**/**G’** were terminal sugar units, in full accordance with the linkage analysis data. The core OS sequence was then achieved based on *inter*-residue NOE correlations detected in the ROESY spectrum (Figure S4), complemented by long-range correlations attained from the HMBC spectrum (Figure 1B). Briefly, starting from Kdo unit (**K**), this was substituted at the O-5 position by α-D-Man **B**/**B’**, as indicated by the long-range correlation between the H-1 signal of **B**/**B’** and the C-5 signal of **K** (Figure 1B). α-D-Man **B**/**B’** was in turn substituted at O-2 by β-D-Glc **G**/**G’** and at O-6 by β-D-Glc **H**, as indicated by the NOE correlation between H-1 of **G**/**G’** and H-2 of **B**/**B’** and between H-1 of **H** and H-6 of **B**/**B’** (Figure S4), as well as by the related HMBC correlations (Figure 1B). The latter residue **H** showed to bear α-D-Glc **D** at its O-6 position, as proven by the HMBC correlation between anomeric carbon atom of **D** with H-6 of **H**. The latter α-D-Glc **D** in turn carried the α-D-Man **C** at its O-2 position, whereas **C** was substituted at its O-2 position by the terminal α-D-Man **E**, as indicated by the related NOE contacts and HMBC correlations (Figures 1B and S4). Altogether, NMR data and compositional analyses, showed that the core OS of *S. copri* R-LPS was a heptasaccharide decorated by a *P*EtN and a phosphate and composed of one Kdo, two β-D-Glc, three α-D-Man, and one α-D-Glc, as sketched in Figure 1C,D.

**Supporting note 3.**

**ESI-MS and MS/MS characterization of the R-LPS from *S. copri* DSM 18205 R-LPS**

The intact R-LPS sample was analyzed by direct infusion using negative-ion mode ESI-MS (Figure 2A). A complex distribution of ions corresponding to both intact R-LPS species and their characteristic fragment ions related to the lipid A and core OS was evidenced. Intact R-LPS molecules were predominantly observed in the triply ([M-3H]^3−^) and doubly ([M-2H]^2−^) charged states, with major signals detected at *m/z* 1022.767 and at *m/z* 1534.609 (3070.582 Da) (Figure 2A,B, Table S3), consistent with the full R-LPS structure comprising a *mono*-phosphorylated penta-acylated lipid A moiety (1675.2 Da) linked to a core OS consisting of six hexoses (Hex), one Kdo, one phosphate group, and one 2-aminoethyl phosphate (*P*EtN), thus in agreement with NMR analysis. A magnified view of the spectrum (Figure 2B) in the *m/z* 1360–1710 range provided insights into the heterogeneity of lipid A and R-LPS species. Multiple series of [M-2H]^2−^ ions corresponding to R-LPS variants were observed, differing in acylation pattern, phosphorylation state, and presence of sodium adducts (Table S3). Moreover, [M-2H]^2−^ ions attributed to R-LPS species decorated by four acyl chains and one or two phosphates on the lipid A were clearly identified in the mass range *m/z* 1400-1500 (Table S3). Additionally, [M-H]^−^ ions at *m/z* 1660.024, 1674.035, 1688.046 and 1702.057 were identified as *mono*-phosphorylated penta-acylated lipid A species (Figures 2A,B). A minor population of [M–3H]^3-^ ions was also detected at approximately *m/z* 1076.775 (3232.665 Da) (Figure 2A) and was assigned to R-LPS species carrying an additional Hex residue not identified by NMR, thereby suggesting minor heterogeneity within the core OS structure. Structural assignments were based on accurate mass measurement in MS and MS/MS analysis using collision-induced dissociation (CID) on selected precursor ions. The huge heterogeneity of ion species and the broad distribution of charge states complicated MS/MS analysis, as overlapping isotopic distribution made it difficult to isolate minor precursor ions for targeted fragmentation. However, MS/MS analysis was performed on some of the most intense ion species detected. Examples of MS/MS spectra have been reported in Figures 3, S6-S7, where some of the cross-ring fragmentations were described and for isomeric fragment ions, only one of all possible annotations is reported per fragment ion. When feasible, traveling wave ion mobility separation (TWIMS) was also employed to obtain simplified mass spectra. After quadrupole isolation, the precursor ion was subjected to CID in the trap region by applying fixed collision energy and then filtered by ion mobility to obtain mass spectrum. Specifically, fragmentation and separation by ion mobility of the [M-3H]^3−^ precursor ion at *m/z* 1022.767 (Figure S6) led to clear visualization of the lipid A and core OS related fragments. As example, clear B- and C-type ions,^[24]^ related to core OS, and generated by the loss of a phosphate (*m/z* 1296.252) and both a phosphate and the neutral loss of CO_2_ from Kdo (*m/z* 1252.268 and 1270.274) were clearly visualized (Figure S6). Finally, Figure 2A also revealed a series of prominent signals within the *m/z* 757.432–778.451 range, consistent with [M-2H]^2-^ ions corresponding to tetra-acylated lipid A species decorated by two phosphate groups. To gain deeper insights into this structural heterogeneity and to pinpoint the exact position of the second phosphate group on the glucosamine disaccharide, we carried out negative-ion MS/MS analysis on these selected precursor ions. As an example, in Figure S8 is reported the negative-ion MS/MS analysis of precursor [M-2H]^2^ ion at *m/z* 764.438 (1529.016 Da) where the presence of two phosphate groups was immediately suggested by the detection of diagnostic fragment ions at *m/z* 176.968 and *m/z* 158.959, corresponding to [H_3_P_2_O_7_]⁻ and [HP_2_O_6_]⁻, respectively. These pyrophosphate-related ions are commonly observed in *bis*-phosphorylated lipid A species and can arise regardless of the presence of a true pyrophosphate linkage; by contrast they are absent in MS/MS spectra of *mono*-phosphorylated lipid A, where only monophosphate-related fragment ions at *m/z* 96.992 ([H_3_PO_4_]⁻) and *m/z* 78.978 ([PO_3_]⁻) could be detected. Moreover, fragmentation of the [M−2H]²⁻ ion at *m/z* 764.4 (1528 Da; Figure S7) generated diagnostic fragment ions of Y_1_ and C_2_ types at *m/z* 766.478 and *m/z* 778.899, respectively. These ions supported the presence of two 16:0(3-OH) chains and one phosphate on the reducing glucosamine, and one 17:0(3-OH), one 17:0, and one phosphate on the non-reducing glucosamine, thereby assigning the second phosphate to the latter unit.

**Supporting note 4.**

**Bioinformatic analysis of genes encoding for *S. copri* DSM 18205 R-LPS biosynthesis**

Our genome analysis predicted a total of 2926 protein-coding sequences and 1765 transcriptional units, including both polycistronic operons and single-genes transcripts. Using the predicted protein sequences, we performed a functional interaction analysis with STRING. Focusing specifically on LPS biosynthesis, we selected interaction networks associated with the Gene Ontology terms *GO:0009103* (lipopolysaccharide biosynthetic process), *GO:0009245* (lipid A biosynthetic process), and *GO:1903509* (liposaccharide metabolic process), as well as the KEGG pathway *map00540* (lipopolysaccharide biosynthesis). This approach yielded two interaction networks comprising 24 proteins (Figure S9, Table S4) and 29 proteins (Table S4), respectively, based on GO and KEGG annotations (STRING v12: https://version-12-0.string-db.org/organism/STRG0A75DLZ). Both networks displayed significant protein–protein interaction (PPI) enrichment, with *p*-values < 1.0e^−16^, indicating that these proteins interact more frequently than expected by chance for random protein sets of comparable size and degree distribution. This suggests that the proteins are functionally associated as a biological group, i.e. they are likely to participate in a coordinated biosynthetic pathway, reinforcing the idea that they collectively contribute to the assembly and regulation of the R-LPS structure in *S. copri*. Operon prediction using the Operon Mapper web server indicated that the genes encoding these proteins are distributed across multiple operons or are transcribed individually (Figure S10). In total, 82 proteins were predicted to be encoded and their Pfam domains were annotated and visualized (Figure S11). Then, we examined the genomic context associated with *S. copri* R-LPS biosynthesis.

The biosynthesis of lipid A, the membrane-anchoring moiety of LPS, is classically regulated through nine conserved enzymatic steps known as the Raetz pathway.^[26]^ In our analysis, we identified homologs for all enzymes of the Raetz pathway, except for the secondary acyltransferases, for which only one homolog (LK433_RS02745) was detected. This gene showed similarity to *lpxL*, suggesting that *S. copri* likely synthesizes a penta-acylated lipid A rather than a hexa-acylated form. This finding is consistent with our MS data and aligns with known traits of other Bacteroidetes species.^[9,14,27]^ Interestingly, our analysis revealed the presence of two *lpxA* homologs, LK433_RS13255 and LK433_RS06380, predicted to encode two LpxA-like proteins WP_006849066.1 (ScLpxA_2) and WP_006847229.1 (ScLpxA) that exhibited 30.35% and 43.89% sequence identity to *E. coli* LpxA (EcLpxA) respectively (Table S5). Moreover, the two homologs shared the same orthologous group (COG1043) and Pfam domains (PF13720 and PF00132) commonly associated with LpxA function and were also integrated in the functional networks described above. Domain analysis using InterProScan predicted that ScLpxA_2 WP_006849066.1 harbors a UDP-*N*-acetylglucosamine (GlcNAc) *O*-acyltransferase domain between residues 176-257, while the remaining of the protein comprises six hexapeptide repeats, a feature typical of bacterial transferases. Catalytic residues were predicted at positions 121 and 122, with H121 (histidine) acting as a proton acceptor and D122 (aspartic acid) contributing to the basicity of the active site. Similarly, *ScLpxA* was predicted to contain the same acyltransferase domain (residues 175-255) and hexapeptide repeats but also exhibited thirteen additional conserved residues (Q69, K72, S95, H117, H120, D121, E139, H155, Q156, G168, N193, R199, R200). Among these, H120 and D121 are predicted to perform the same catalytic roles as in ScLpxA_2. Notably, these conserved histidine and aspartic acid residues are also found in the well-characterized EcLpxA, where the histidine acts as the catalytic base facilitating the nucleophilic attack by the 3-OH group of GlcNAc, enabling the transfer of the *R*-3-hydroxymyristoyl chain [14:0(3-OH)] from its acyl carrier protein donor.^[29]^ Moreover, *Clustal Omega* was used to align the protein sequences of the two *S. copri* LpxA homologs to the sequence of the previously characterized LpxA from *Bacteroides fragilis* (BfLpxA).^[68]^ This analysis confirmed that ScLpxA retains all residues known to hydrogen-bond with the UDP-GlcNAc substrate in both BfLpxA and EcLpxA. By contrast, ScLpxA_2 shares these residues with two exceptions, i.e. substitutions at the positions corresponding to Q154 and R197 of BfLpxA, suggesting differences in the ability to bind the substrate (data not shown). To further assess structural conservation, we superimposed the AlphaFold3 predicted^[30]^ 3D models of ScLpxA and ScLpxA_2 onto the crystallographic structure of EcLpxA (PDB ID: 1LXA)^[69]^ using PyMOL. For comparison, the crystal structure of *Bf*LpxA (PDB ID: 4R36)^[66]^ was also aligned with EcLpxA. The resulting root-mean-square deviation (r.m.s.d.) values ranged from 0.666 Å to 0.773 Å, with key catalytic residues displaying substantial spatial overlap, supporting the reliability of the predicted models and validating the functional annotations (Figure S12O).

To further investigate the two LpxA homologs identified in *S. copri*, we employed AlphaFold3 to predict their possible oligomeric states. Specifically, we modelled both homotrimeric complexes (ScLpxA × 3 and ScLpxA_2 × 3), as well as the two hypothetical heterotrimer composed of either two ScLpxA subunits and one ScLpxA _2 subunit or two ScLpxA_2 subunits and one ScLpxA subunit. The homotrimer formed by ScLpxA_2 displayed high model confidence, with ipTM and pTM scores of 0.93 and 0.94, respectively. The predicted local distance difference test (pLDDT) scores were consistently above 90 for most regions of the structure, except for residues 70-76, 102, 220-223, and 259-260 in each monomer, where pLDDT values dropped to the 70-90 range, indicating moderate confidence. Similarly, the ScLpxA homotrimer showed even higher model confidence, with ipTM and pTM scores of 0.95 and 0.96. As in the previous case, pLDDT scores were >90 throughout the structure, except at residue 221 in each monomer, which showed a slight drop in confidence (pLDDT 70–90). Both heterotrimeric complexes yielded slightly lower ipTM (0.87 and 0.86) and pTM (0.90 both) values. Although the overall pLDDT scores remained high (>90), local regions with slightly reduced confidence (70 < pLDDT < 90) were also observed, mirroring the homotrimeric profiles. To gain further insights into subunit interactions and potential interface stability, all four AlphaFold3-generated models were submitted to AlphaBridge.^[31]^ This analysis produced refined structures and identified interface residues likely involved in trimer formation (Figure S12). The two homotrimeric complexes consistently showed high interface confidence, with ipTM, contact ipTM, and AlphaBridge scores around 0.9. In contrast, both the heterotrimer exhibited significantly lower contact ipTM and AlphaBridge scores, suggesting weaker or less stable interfaces. Notably, increasing the stringency for interface detection markedly altered the interaction profiles of the two possible heterotrimers, while predictions for the homotrimers remained stable, further supporting their robustness. Whether these two LpxA orthologs can form stable heterotrimeric assemblies in vivo remains to be experimentally validated. However, their relatively low sequence identity (45.06%), divergence in conserved residues, and differences in trimeric interface architecture suggest they may have distinct substrate specificities in their homotrimeric forms. Of note, comparative genomic analysis revealed that other Bacteroidetes, including *Bacteroides thetaiotaomicron*, *B*. *fragilis* and *B. vulgatus*, also encode two *lpxA* homologs (Table S5). While *E. coli* typically produces less heterogenous lipid A species, these *Bacteroides* species, like *S. copri*, generate a range of structurally distinct lipid A molecules. This observation supports the hypothesis that lipid A heterogeneity may, at least in part, arise from the functional diversification of LpxA enzymes, each capable of incorporating different acyl chains at the 3-OH position of glucosamine. Therefore, while these trimeric assemblies remain to be experimentally confirmed, they may offer a molecular explanation for how *S. copri* fine-tunes the chemical composition of its LPS lipid A, potentially influencing host immune recognition.

To the best of our knowledge, no structural or genetic information is currently available regarding the core OS region of *S. copri* LPS. However, in other Gram-negative bacteria, such as *E. coli*, *B. thetaiotaomicron*, and *P. aeruginosa*, genes involved in core OS biosynthesis have been well characterized.^[70,71]^ Based on this knowledge, and supported by our genomic and functional network data, we identified two genomic regions in *S. copri* as likely candidates for core OS biosynthesis: from LK433_RS02390 to LK433_RS02455 (including operon 271, Figure S10), and from LK433_RS02700 to LK433_RS02755 (including operon 309, Figure S10). The first region includes three glycosyltransferases of the GT2 family, one mannosyltransferase, and a GT9-family glycosyltransferase, predicted to act as heptosyltransferase. Notably, both the mannosyltransferase (WP_006848232.1) and one GT2 enzyme (WP_006848234.1) were found to be involved in our LPS-related STRING networks and are encoded within operon 271, which includes all glycosyltransferases in the region except the GT9 enzyme. Considering that our chemical analysis revealed the presence of mannose and glucose residues, but no heptoses, in the R-LPS structure, the GT9 gene might be regulated independently from the others. This scenario is consistent with observations in *B. thetaiotaomicron* and *B. vulgatus*, which harbor GT9 genes homologous to *E. coli* heptosyltransferases, yet do not incorporate heptoses into their LPS. The second region (operon 309) contains LK433_RS02750, encoding a protein (WP_006848119.1) assigned to COG1132, sharing both COG classification and 36.64% sequence identity (BLASTP) with multiple *E. coli* MsbA proteins. This region also includes a predicted *lpxL* homolog (LK433_RS02750) and one of the two LPS kinases discussed below. Additionally, two putative glycosyltransferases, GT113 (LK433_RS02730) and GT90 (LK433_RS02725), are encoded within the operon. Upstream of the cluster, further glycosyltransferase candidates were identified: a GT4 family enzyme (LK433_RS02720), another mannosyltransferase predicted to belong to the GT32 family, and the second LPS kinase (LK433_RS02705). Given that genes within operons are typically co-regulated, it is likely that the glycosyltransferase genes LK433_RS02730 and LK433_RS02725 are responsible for the addition of sugar residues to the R-LPS. The additional two glycosyltransferases, upstream of the operon, may also participate in core OS assembly: one is predicted to act as a mannosyltransferase, while the GT4-family enzyme could plausibly catalyze the addition of either glucose or mannose, based on established activities within the CAZy GT4 family. According to function prediction we can also speculate that the Kdo is added to the lipid A by the protein encoded by the gene LK433_RS00645, a putative Kdo transferase.

Finally, in addition to the core OS biosynthetic enzymes, we also investigated genes potentially involved in non-carbohydrate modifications (i.e. phosphate and *P*EtN in the case of *S. copri*). Among these, two genes, LK433_RS03400 and LK433_RS06640, were identified within both functional networks and were predicted to encode proteins homologous to *eptA* and *eptB*, which catalyze the addition of PEtN to lipid A and Kdo residues, respectively. Two other genes, LK433_RS10260 and LK433_RS10015, were classified within the same orthologous group (COG0671) as *lpxE*, *lpxT*, and *lpxF*, enzymes known to modulate lipid A phosphorylation. Based on both orthology and network analysis, these two predicted phosphatases may participate in the addition or removal of phosphate groups on the lipid A backbone. Conversely, LK433_RS02735 and LK433_RS02705 were annotated as encoding members of the LPS kinase family (WaaP-like kinases), which are typically responsible for the phosphorylation of the inner core region of LPS. Collectively, these predicted genes encode enzymes that may contribute to the extensive chemical heterogeneity observed in *S. copri* lipid A, not only through acylation pattern diversity but also via phosphorylation and *P*EtN modifications.

**Supporting References**

1. S. H. Duncan, A. Barcenilla, C. S. Stewart, S. E. Pryde, H. J. Flint, *Appl. Environ*. Microbiol. **2002**, *68*, 5186–5190.
2. C. Galanos, O. Luderitz, O. Westphal, *Eur. J. Biochem.* **1969**, *9*, 245–249.
3. M. Hirschfeld, Y. Ma, J. H. Weis, S. N. Vogel, J. J. Weis, *J. Immunol.* **2000**, *165*, 618–622.
4. K. Leontein, *Methods Carbohydr. Chem.* **1978**, *62*, 359–362.
5. E. T. Rietschel, *Eur. J. Biochem*., **1976**, *64*, 423–428
6. E. G. Bligh, W. J. Dyer, *Can. J. Biochem. Physiol.* **1959**, *37*, 911–917.
7. A. S. Stern, K. B. Li, J. C. Hoch, *J. Am. Chem. Soc.* **2002**, *124*, 1982–1993.
8. A. Ceroni, K. Maass, H. Geyer, R. Geyer, A. Dell, S. M. Haslam, *J. Proteome Res*. **2008**, *7*, 1650-1659.
9. D. Damerell, A. Ceroni, K. Maass, R. Ranzinger, A. Dell, S. M. Haslam, *Methods Mol. Biol*. **2015**, *1273*, 3-15.
10. G. Larrouy-Maumus, A. Clements, A. Filloux, R. R. McCarthy, S. Mostowy, *J. Microbiol. Methods* **2016**, *120*, 68–71.
11. B. Taboada, K. Estrada, R. Ciria, E. Merino, *Bioinformatics* **2018**, *34*, 4118–4120.
12. M. Blum, A. Andreeva, L. C. Florentino, S. R. Chuguransky, T. Grego, E. Hobbs, B. L. Pinto, A. Orr, T. Paysan-Lafosse, I. Ponamareva, G. A. Salazar, N. Bordin, P. Bork, A. Bridge, L. Colwell, J. Gough, D. H. Haft, I. Letunic, F. Llinares-López, A. Marchler-Bauer, L. Meng-Papaxanthos, H. Mi, D. A. Natale, C. A. Orengo, A. P. Pandurangan, D. Piovesan, C. Rivoire, C. J. A. Sigrist, N. Thanki, F. Thibaud-Nissen, P. D. Thomas, S. C. E. Tosatto, C. H. Wu, A. Bateman, *Nucleic Acids Res.* **2025**, *53*, D444–D456.
13. C. Chen, Y. Wu, J. Li, X. Wang, Z. Zeng, J. Xu, Y. Liu, J. Feng, H. Chen, Y. He, R. Xia, *Mol. Plant* **2023**, *16*, 1733–1742.
14. J. Zheng, Q. Ge, Y. Yan, X. Zhang, L. Huang, Y. Yin, *Nucleic Acids Res.* **2023**, *51*, W115–W121.
15. V. Zulkower, S. Rosser, *Bioinformatics* **2020**, *36*, 4350–4352.
16. H. M. Berman, J. Westbrook, Z. Feng, G. Gilliland, T. N. Bhat, H. Weissig, I. N. Shindyalov, P. E. Bourne, *Nucleic Acids Res.* **2000**, *28*, 235–242.
17. Schrödinger, L. L. C., *The PyMOL Molecular Graphics System*, Version 1.3r1, 2010, <http://pymol.org/citing>
18. G. I. Birnbaum, *J. Carbohydr. Chem*. **1987**, *6*, 17−39
19. A. Ngo, K. T. Fong, D. L. Cox, X. Chen, A. J. Fishe**r**, *Acta Crystallogr. D Biol. Crystallogr.* **2015**, *71*, 1068–1076.
20. C. R. Raetz, S. L. Roderick, *Science* **1995**, *270*, 997–1000.
21. K. Amor, D. E. Heinrichs, E. Frirdich, K. Ziebell, R. P. Johnson, C. Whitfield, *Infect. Immun.* **2000**, *68*, 1116–1124.
22. J. S. Lam, V. L. Taylor, S. T. Islam, Y. Hao, D. Kocíncová, *Front. Microbiol.* **2011**, *2*, 118.
